# Intradental mechano-nociceptors serve as sentinels that prevent tooth damage

**DOI:** 10.1016/j.celrep.2025.116017

**Published:** 2025-07-16

**Authors:** Elizabeth A. Ronan, Akash R. Gandhi, Karin H. Uchima Koecklin, Yujia Hu, Shuhao Wan, Brian S.C. Constantinescu, Mak E. Guenther, Maximilian Nagel, Ling-Yu Liu, Aditi Jha, Leen Dakhilalla, Kaitlyn J. Blumberg, Isaac T. Berthaume, Tomer Stern, Kevin P. Pipe, Bing Ye, Peng Li, Joshua J. Emrick

**Affiliations:** 1 Department of Biologic and Materials Sciences, University of Michigan, Ann Arbor, MI 48109, USA; 2 Life Sciences Institute, University of Michigan, Ann Arbor, MI 48109, USA; 3 Department of Molecular and Integrative Physiology, University of Michigan, Ann Arbor, MI 48109, USA; 4 Department of Mechanical Engineering, University of Michigan, Ann Arbor, MI 48109, USA; 5 Sensory Cells and Circuits Section, National Center for Complementary and Integrative Health, Bethesda, MD 20892, USA; 6 Department of Cell and Developmental Biology, University of Michigan, Ann Arbor, MI 48109, USA; 7 Lead contact

## Abstract

The trigeminal sensory neurons that innervate the tooth’s vital interior—intradental neurons—are expected to drive severe pain, yet their contribution to healthy tooth sensation has not been explored. Here, we uncover a role for myelinated high-threshold mechano-nociceptors (intradental HTMRs) in tooth protection using *in vivo* Ca^2+^ imaging, opto-/chemogenetics, and the AI-driven behavioral analysis tool LabGym. Intradental HTMRs innervate the inner dentin through overlapping receptive fields and respond as the external structures of the tooth are damaged in the absence of either PIEZO2 or Na_v_1.8. Whereas chemogenetic activation of intradental HTMRs results in a pain phenotype marked by facial and postural changes, their transient optogenetic activation triggers a rapid, jaw-opening reflex via contraction of the digastric muscle. Our work indicates that intradental HTMRs not only trigger pain but also protect the teeth by initiating a reflexive movement of the jaws when the teeth experience damage during chewing.

## In brief

Ronan et al. explore the function of trigeminal sensory neurons innervating the inner tooth—herein, intradental neurons. They determine these specialized cells respond to damage of the enamel and initiate both pain and a jaw-opening reflex. Their work shifts our perspective of tooth innervation from pain producing to protective.

## Graphical Abstract

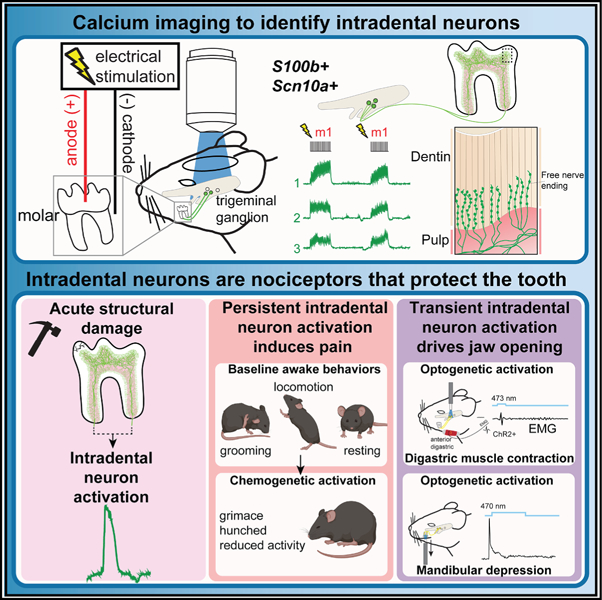

## INTRODUCTION

Mammals instinctively use their teeth for mechanical behaviors including chewing to initiate digestion and biting for prey capture and self-defense. The protection and maintenance of enamel and teeth are essential for mammals with teeth that do not continually regenerate. In humans, loss of teeth is associated with poorer diet and nutritional status^1^ as well as early mortality.^2^ The somatosensory neurons within the trigeminal ganglion (TG) extend processes into the oral and craniofacial tissues, including the inner tooth. Broadly, activation of primary sensory cells and their downstream neuronal targets produces outputs including perceptions (e.g., touch, temperature, body position, and pain) and sensorimotor reflexes.^3,4^ While we assume that the activation of tooth innervation is the source of substantial pain,^5–7^ whether tooth-innervating somatosensory neurons (hereafter, intradental neurons) might contribute to the protection of tooth structure in health has not been explored.

The tooth comprises external mineralized layers (i.e., outer-most enamel then dentin) and an internal soft tissue (i.e., dental pulp). Historically, intradental innervation is thought to arise from both myelinated (Aβ/Aδ) and unmyelinated (C-type) trigeminal sensory neurons. However, myelinated intradental neurons pre-dominate based on immunohistochemical labeling,^8^ and transcriptomic analyses indicating that the majority of intradental neurons express *S100b*, a marker of myelinated somatosensory neurons.^9,10^ While electrophysiological experiments have demonstrated that fibers with conduction velocities in the Aδ range spike in extreme conditions when the inner tooth has been exposed,^11^ linking the molecular identity of intradental neurons with their capacity to respond in an intact tooth has not been explored. The combination of transcriptomic analyses with *in vivo* functional imaging provides a new ability to relate molecular identity with cell function.^3,9,12–16^ Indeed, these tools have revealed the basis for touch sensation in the skin,^10,17^ and provide great potential for defining the role of intradental neurons.

Here, we develop a strategy to identify intradental neurons in the TG without damaging the tooth, then monitor their response profiles to mechanical stimulation. We determine most responding neurons are large diameter and co-express transcriptomic markers of myelinated high-threshold mechano-nociceptors (HTMRs). These intradental neurons do not encode any direct forces applied to the intact outer tooth but are activated once the dentin has been exposed. Intradental terminal endings extend into the inner dentin providing an anatomical basis for their observed dentin-response profile. Importantly, we show that intradental neurons collectively respond to superficial damage to the tooth independent of *Piezo2* or *Scn10a*. We find that chemogenetic activation of intradental HTMRs elicits orofacial grimace and hunched posture that is indicative of pain. Additionally, we demonstrate optogenetic activation of intradental HTMRs evokes a rapid jaw-opening response via digastric muscle contractions that withdraws opposing teeth from contact. This work not only provides a cellular target for addressing acute tooth pain, but also provides a new perspective on intradental neuron function as sentinels that monitor for mechanical threats and initiate a reflex to protect the teeth.

## RESULTS

### *In vivo* identification of large-diameter intradental neurons through electrical stimulation of the intact tooth

**Figure 1.**
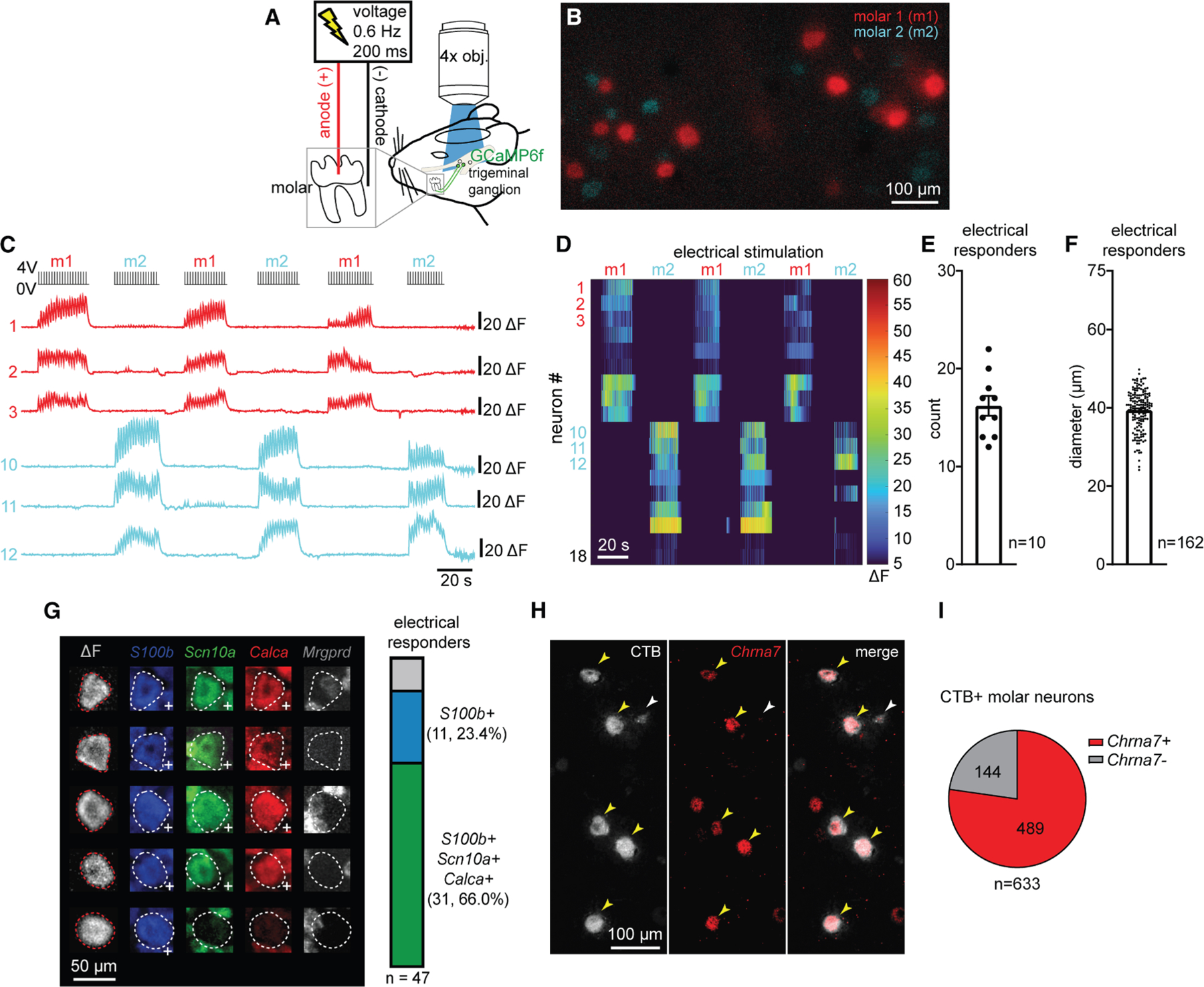
Functional imaging of intradental neurons reveals a large diameter population of putative myelinated HTMRs (A) Schematic *in vivo* recording of the TG. Intradental neurons were identified using low-voltage pulsing (4 V, 0.6 Hz, 200 ms) of individual mandibular molars. Data in (A)–(F) were obtained from adult Ai95(RCL-GCaMP6f)-D (Ai95D) mice injected postnatally (P0-P3) with AAV9-Cre. (B–D) Electrical stimulation identified intradental neurons innervating a single molar. (B) Imaging field showing merged single frames of Ca^2+^ response (ΔF). Neurons from stimulation of either molar 1 (m1, red) or molar 2 (m2, cyan). Scale bar, 100 μm. (C) Example traces of GCaMP6f from m1 (red) and m2 (cyan) intradental neurons. Numbers correspond to (B). Vertical scale bars, 20 ΔF. Horizonal scale bar, 5 s. (D) Example heatmap from single TG showing responses of 18 intradental neurons. Scale bar, 20 s. Traces in (C) are neurons from (D). (E and F) (E) Bar graph showing mean neurons per trial or (F) mean diameter of neurons responding to ≥2 voltage pulses (=intradental neurons). *n* = 162 neurons from 10 mice. Plotted individual data points represent neurons in separate imaging trials. Error bars indicate the SEM. (G) Alignment of calcium responses with multiplexed whole-mount *in situ* hybridization (ISH) enables molecular classification of intradental neurons. Data were obtained from Ai95(RCL-GCaMP6f)-D (Ai95D) mice injected postnatally (P0-P3) with AAV9-hSyn1-mCherry-2A-iCre. mCherry expression was used to align fluorescence responses from Ca^2+^ imaging with ISH images. (Left) Multiplexed whole-mount ISH staining following alignment to GCaMP6f fluorescence. Probes: s100 calcium-binding protein B (*S100b*, blue), α-Nav1.8 (*Scn10a*, green), calcitonin gene-related peptide (CGRP; *Calca*, red), Mas-related G protein-coupled receptor member D (*Mrgprd*, white). (+), positive staining. Scale bar, 50 μm. (Right) Vertical bar (parts of a whole) indicates number and percentage of intradental neurons expressing selected genes. *n* = 5 mice, comprising 47 intradental neurons. (H and I) Representative image of retrograde-labeled intradental neurons (CTB-AF647) and ISH for *Chrna7*. (H) Left panel: CTB labeling; middle panel: *Chrna7* expression; right panel: merge. Scale bar, 100 μm. Yellow arrowheads, intradental neurons co-positive for CTB-AF647 and *Chrna7*; white arrowheads, cells only positive for CTB. (I) Pie chart demonstrating co-expression. *n* = 4 mice, comprising 633 retrograde-labeled intradental neurons. See also Figure S1 and Video S1.

To evaluate the function of intradental neurons, we adapted an *in vivo* trigeminal Ca^2+^ imaging technique in mice to visualize real-time activity of sensory neurons^3,18^ while stimulating individual molars (Figures 1A–1F and S1A–S1C). Unbiased labeling of trigeminal sensory neurons was achieved using neonatal AAV-Cre injections in Ai95D (Rosa-LSL-GCaMP6f) mice as previously described.^10,19^ This approach broadly labeled TG neurons across all cell diameters (range = 7–52 μm, mean = 28 μm) with a ∼50% transduction efficiency (Figure S1D). Past work in large mammals indicates that electrical stimulation applied to exposed dentin can elicit single unit responses within the alveolar nerve.^20^ Furthermore, the electric pulp test is routinely used in clinical dentistry to assess tooth sensation in human patients. Thus, we reasoned that low voltage pulses delivered to the occlusal surface of individual intact molar teeth may reveal associated intradental neurons. Electrical stimulation of molar 1 or molar 2 (2–4 V, 0.6 Hz, 200 ms, Figure 1A, see STAR Methods) resulted in stereotyped phasic, transient Ca^2+^ responses from separate groups of trigeminal sensory neurons (Figures 1B–1D; Video S1) indicating discrete sensory inputs from each tooth. We identified pulsing neurons (12–22 neurons, *n* = 10, Figure 1E) with large diameters (mean = 39.4 ± 0.4 μm, *n* = 162 neurons, Figure 1F) consistent with the upper range of cell diameters of Aδ-myelinated sensory neurons. These neurons also responded to another clinical assay for human tooth pulp sensitivity^21^ (Endo-Ice-mediated cold, Figure S1E). To confirm that electrical stimulation preferentially activates intradental neurons, we performed retrograde labeling (CTB-AF647) to ipsilateral mandibular molars 1 and 2^22^ prior to Ca^2+^ imaging. Indeed, the majority of responding neurons were labeled by CTB (46/51, *n* = 4 mice, Figure S1F), indicating that electrical stimulation is a reliable method for identifying intradental neurons. Taken together, we have established an *in vivo* method to identify intradental neurons within an intact tooth enabling subsequent molecular and functional evaluation.

### Intradental neurons express markers of putative HTMRs

We next sought to determine the molecular identity of responding intradental neurons. To this end, we performed Ca^2+^ imaging followed by post-hoc *in situ* hybridization (ISH) of whole-mount dissected TG.^10^ We reasoned that an initial panel of probes (*S100b*, *Scn10a*, *Calca*, and *Mrgprd*)^10,22^ would allow us to differentiate whether intradental neurons represent one or more broad mechanoreceptor subtypes. We again relied on neonatal AAV injection to induce simultaneous GCaMP6f and mCherry expression in trigeminal sensory neurons (Figure S1D). mCherry fluorescence and ISH provided reliable guideposts (Figure S1G) to align ISH staining (Figure S1H) with fluorescence responses from Ca^2+^ imaging. These alignments revealed that some responding intradental neurons expressed *S100b* alone (11/47) but most expressed *S100b*, *Scn10a*, and *Calca* (31/47), which was consistent with our prior *in vitro* characterization indicating that the majority represent nociceptive large diameter, myelinated HTMRs.^10^ Conversely, intradental neurons lacked expression of *Mrgprd* (1/47), a marker of unmyelinated C-type HTMRs^10^ (Figure 1G). To further confirm our molecular characterization, Ca^2+^ imaging of *S100b*+ trigeminal sensory neurons (*S100b*-Cre; Ai95D) enabled identification of intradental neurons (Figures S1I and S1J) and indicated that our electrical stimulation paradigm is capable of capturing >75% of all intradental neurons captured by retrograde labeling with CTB (range = 70%–87%, 83/111, *n* = 4, Figure S1K). Together these results demonstrate that intradental neurons primarily represent myelinated nociceptive HTMRs.

Recent studies revealed that HTMRs in the dorsal root ganglia (DRG) can be further differentiated by the expression of select transcriptional markers,^23^ which may apply to myelinated HTMRs of the TG.^24^ Specifically, *S100b*+/*Scn10a*+ myelinated HTMRs represent two groups that are distinguished by the expression of *Bmpr1b*, *Chrna7*, or *Smr2* (Figure S1L). To evaluate if intradental neurons represent one (or more) of these molecularly defined subclasses of HTMRs, we first marked intradental neurons using retrograde tracing (CTB-AF647) from the ipsilateral molar teeth, then performed ISH to determine coexpression of *Bmpr1b*, *Chrna7*, or *Smr2*. As predicted, we found that *Bmpr1b*, *Chrna7*, and *Smr2* are expressed within subsets of TG sensory neurons (Figures 1H and S1M). Importantly, we determined that *Chrna7* (489/633, ∼77%, *n* = 4 mice, Figure 1I) was found in the majority of CTB+ intradental neurons. Few CTB+ neurons expressed either *Smr2* or *Bmpr1b* (*Smr2*: 5/229, ∼2%, *n* = 3 mice; *Bmpr1b*: 8/102, ∼8%, *n* = 3 mice; Figure S1M). Taken together, these data indicate that intradental neurons are putative myelinated HTMRs marked by *S100b*, *Scn10a*, and *Chrna7* expression.

### Intradental neurons respond to direct force after enamel removal

**Figure 2.**
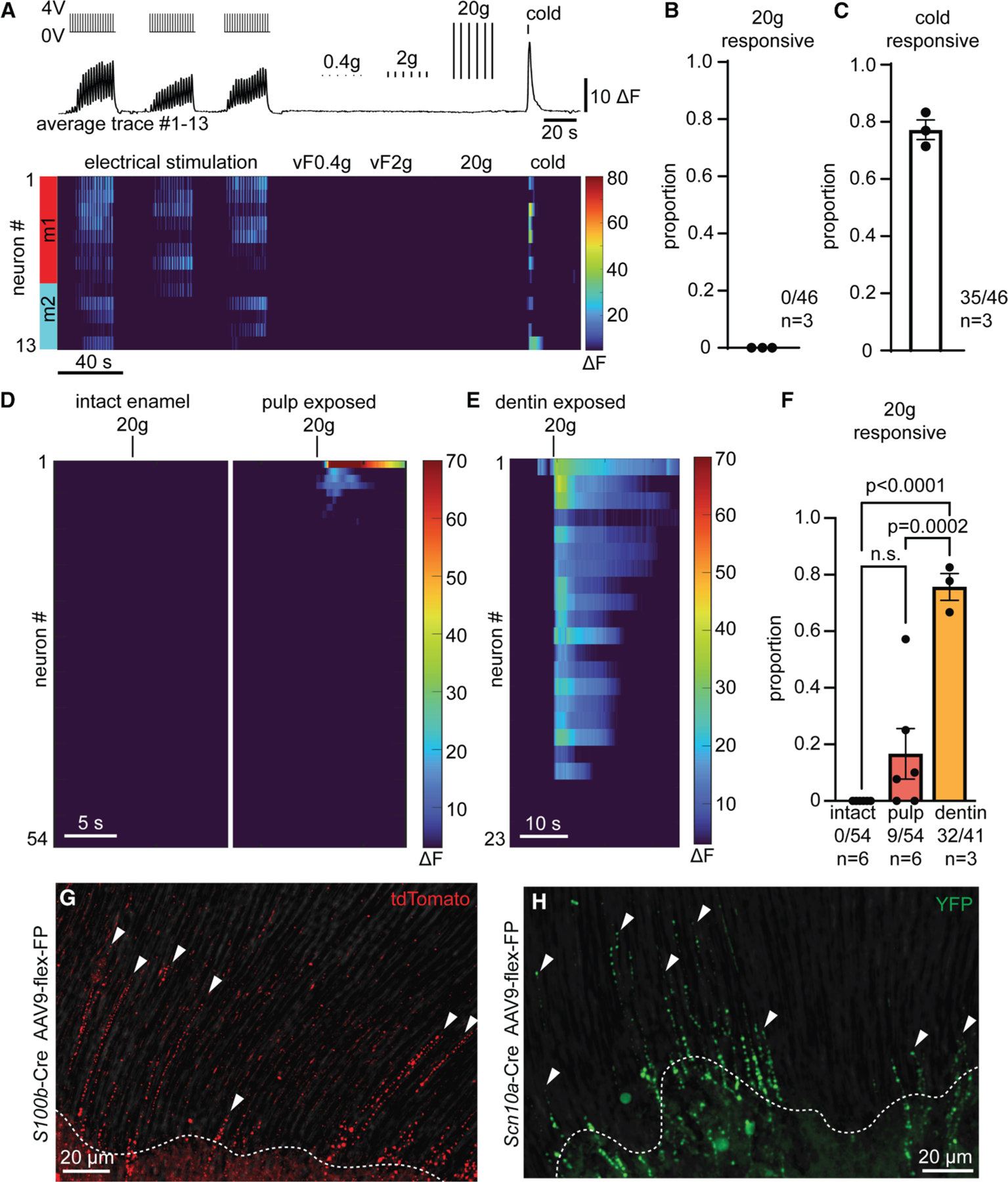
Intradental neurons are HTMRs that encode direct force after enamel removal and innervate the dental pulp and inner dentin (A–C) Intradental neurons do not respond to direct forces applied to the intact tooth. (A) Example trace (top) and associated heatmap (bottom). Stimuli used are indicated above the traces. Vertical scale bar, 10 ΔF. Horizontal scale bars, 20 s (top, trace) or 40 s (bottom, heatmap). (B) Bar graph showing proportion of intradental neurons that respond to direct force. Plotted individual data points represent proportion of force responsive/intradental neurons per trial. Bar shows the mean and error bars indicate the SEM. *n* = 3 mice, comprising 46 cells. (C) Bar graph showing proportion of intradental neurons responding to cold. Plotted individual data points represent the proportion of cold responsive/intradental neurons/trial. Bar shows the mean and error bars indicate the SEM. *n* = 3 mice, comprising 46 cells. Data for (A)–(C) were obtained from Ai95(RCL-GCaMP6f)-D (Ai95D) mice injected postnatally (P0) with AAV9-Cre. (D–F) Intradental neurons respond to direct forces following structural damage of the tooth. (D) Combined heatmap showing response profiles of 54 intradental neurons from 3 mice before (left) and following pulp exposure (right). All neurons represent intradental neurons as determined by electrical stimulation. *De novo* responders were not noted outside of the intradental neurons. Scale bar, 5 s. (E) Example heatmap showing the response of 23 intradental neurons to direct force applied to exposed dentin. Scale bar, 10 s. (F) Bar graph showing proportion of direct force response/intradental neurons. Plotted individual data points represent the proportion of force responsive/intradental neurons per trial. Bar shows the mean and error bars indicate the SEM. *n* = 6 mice for intact enamel, *n* = 6 mice for pulp exposed, *n* = 3 mice for dentin exposed. *p* < 0.001, *p* = 0.002, *p* > 0.05 using one-way ANOVA with Tukey’s correction as indicated with the graph. Data for (D)–(F) obtained from Ai95(RCL-GCaMP6f)-D (Ai95D) mice injected postnatally (P0-P3) with AAV9-Cre. (G) Representative image of immunostained neuronal terminals merged with bright-field image of dentin tubules in demineralized molars from *S100b*-Cre mice receiving TG injection of AAV9-flex-tdTomato for sparse labeling. *n* = 5 mice; Scale bar, 20 μm. (H) Representative image of immunostained neuronal terminals merged with bright-field image of dentin tubules in demineralized molars from *Scn10a*-Cre mice injected using a Cre-dependent AAV to induce sparse labeling of terminals (see STAR Methods). Enamel, a fully mineralized structure, has been removed completely by demineralization. This experiment was repeated in *n* = 4 mice with additional littermate and no primary controls. Scale bar, 20 μm. See also Figure S2.

As intradental neurons express transcriptional markers of HTMRs, we next set out to determine their responses to mechanical forces directed to the intact tooth. We first tested whether intradental neurons respond to a range of innocuous to noxious forces (0.4–20 g). Interestingly, we found that none of the intradental neurons were activated by direct mechanical forces (Figures 2A–2C). Considering exposed dentin is associated with sensitivity in humans in the clinical setting,^25^ we reasoned that intradental neurons might detect mechanical forces in exposed teeth. To test this, we evaluated responses to application of force before and after exposing the dental pulp. As expected, we found that intradental neurons were not activated by the mechanical force on the intact tooth (Figure 2D). In contrast, immediately after the dental pulp was exposed, a portion of intradental neurons now responded to mechanical stimulation (9/54, 17% of electrical responders, Figures 2D and 2F). Furthermore, in teeth where only the dentin was exposed, direct mechanical force now recruited responses from the majority of intradental neurons (32/41, 78% of electrical responders, Figures 2E and 2F). Notably, responses were not due to CD45^+^ immune cell infiltration at this immediate stage (Figures S2A–S2E). From these experiments, we conclude that intradental neurons are HTMRs (intradental HTMRs) that do not contribute to any direct forces applied to the intact tooth but respond to mechanical force after the removal of enamel.

### Intradental HTMRs terminate in the tooth pulp and inner dentin

Our experiments testing direct force on tooth structures suggest the presence of terminal endings of the intradental neurons within the dentin, as mechanical stimulation of exposed dentin recruits their activation in the TG. We applied genetic strategies relying on *S100b* and *Scn10a* to drive fluorescent labeling of intradental HTMRs terminal endings. While *S100b* delineates myelinated somatosensory neurons, it is also broadly expressed in the nervous system as well as non-neuronal peripheral cells. Here, we crossed *S100b*-Cre into a Cre-dependent neuronal-specific reporter strain (*Snap*25-LSL-2A-eGFP) to restrict GFP+ labeling to sensory neurons. We found dense GFP+ innervation of the coronal pulp and parallel GFP+ terminals penetrating to the inner third of the dentin (Figures S2F–S2H). As *Scn10a* expression is highly restricted to sensory neurons, we repeated immunohistochemical experiments in *Scn10a*-Cre; Ai32 mice (*Scn10a*-Cre; LSL-ChR2-YFP). Here, we observed remarkably similar YFP+ innervation patterns within the pulp and inner dentin (Figures S2I and S2J).

While these studies indicate that dentin is uniformly innervated by endings stemming from intradental neurons, the endings of individual neurons could not yet be discerned. We reasoned that sparse labeling of terminal endings^26^ (Figures S2K–S2M) would reveal terminal patterns within the dentin. Here, fluorescently labeled fibers (*S100b*-tdT and *Scn10a*-GFP) were localized to only a portion of the overall dentin, typically within a single pulp horn (Figures 2G and 2H), and exhibited tuft-like patterning that penetrated into non-contiguous dentinal tubules. These findings indicate that single neurons target several tubules and multiple neurons innervate a local region of the inner dentin. With our sparse labeling, we also noted that terminal morphology appeared as a series of puncta running in parallel along the length of dentin tubules (Figures 2G and 2H). Taken together, we establish that intradental HTMRs, as defined by expression of *S100b* and *Scn10a*, contribute intermingled terminals to create dense innervation of the inner dentin.

### Intradental HTMRs function to detect tooth enamel damage

Intradental HTMRs respond to mechanical stimulation following dentin exposure, providing input *after* the inner layers of the tooth have been exposed. We next sought to explore if these neurons also activate *as* molar teeth are damaged. To this end, we monitored responses of trigeminal sensory neurons while simultaneously damaging the enamel surface using a dental bur. Indeed, cutting of the surface enamel evoked responses from most intradental HTMRs (64/70, 91%, *n* = 4, Figures S3A–S3C). Conversely, vibration (50–200 Hz) applied to the tooth evoked almost no activity (2/52, 4%, Figures S3D–S3G) indicating that these cells respond to the damage component of cutting. Taken together, these experiments indicate that intradental HTMRs encode damaging mechanical stimulation of individual teeth.

### Intradental HTMRs detect superficial frictional damage of the tooth surface and encode damage severity

**Figure 3.**
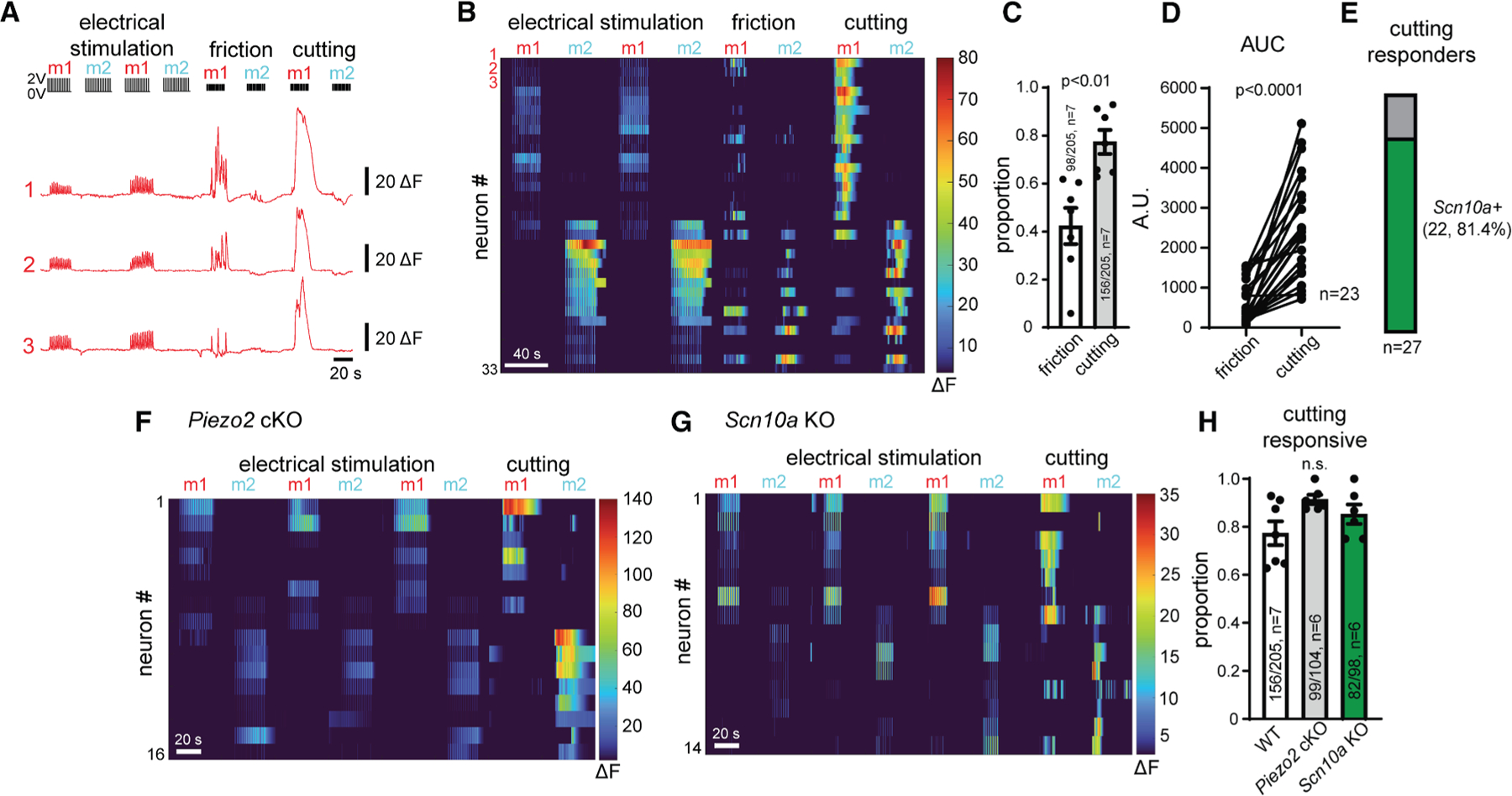
Intradental HTMRs detect enamel damage, independent of *Piezo2* or *Scn10a* (Na_v_1.8) (A–E) Intradental HTMRs respond to cutting and frictional damage of tooth. (A) Example traces showing intradental neuron response to enamel cutting and friction applied to individual molars. Vertical scale bars, 20 ΔF. Horizontal scale bar, 20 s. (B) Example heatmap containing traces from (A). Scale bar, 40 s. (C) Bar graph showing proportion of friction and cutting responsive intradental neurons. Plotted individual data points represent proportion for each trial. Bar shows the mean and error bars indicate the SEM. *n* = 7 mice, comprising 205 total cells, *p* < 0.01, unpaired t test. (D) Line graph depicting magnitude of Ca^2+^ responses of intradental neurons to friction vs. cutting. Individual points represent area under the curve (AUC) during stimulation for each cell. *n* = 23 cells, *p* < 0.0001, paired t test. (E) Vertical bar (parts of a whole) indicates number and percentage of cutting responsive *Scn10a+* neurons (81.4%, 22/27 neurons, *n* = 4 mice). Results in (E) from primary data also used in Figure 1G. (F and G) Example heatmap showing intradental HTMR responses to cutting persist in (F) *Piezo2*-cKO mice or (G) *Scn10a*-KO mice. Scale bars, 20 s. (H) Bar graph depicting the proportion of intradental HTMRs that respond to enamel cutting in wild-type, *Piezo2*-cKO, or *Scn10a*-KO mice. Plotted individual data points represent calculated proportion of enamel cutting responsive/intradental neurons. Bar shows the mean, and error bars indicate the SEM. *n* = 6 or 7 adult mice/genotype. n.s. *p* > 0.05 using one-way ANOVA with Tukey’s correction. See also Figure S3.

The cutting of the exterior tooth produced overt damage of the enamel layer (Figure S3H) and triggered a response from intradental HTMRs. Does the response profile of these neurons provide information about the degree of tooth damage? To this end we sought to introduce a stimulus that creates lesser damage by removing the carbide flutes from the dental bur. This ‘‘fluteless’’ bur produces friction, but not perceptible damage (Figure S3H). Remarkably, friction on the surface of the tooth triggered the activation of almost half of the intradental HTMRs identified by electrical stimulation (98/205, 48%, *n* = 7 mice, Figures 3A–3C). Subsequently, the cutting stimulus evoked responses in these same initial 98 neurons in addition to recruiting responses from 58 additional intradental HTMRs (156/205, 76%, *n* = 7 mice, Figures 3A–3C). Interestingly when examining neurons that responded to both stimuli, friction triggered smaller magnitude responses compared with cutting, suggesting that intensity of activation increases with degree of tooth damage (Figure 3D). Post hoc ISH again confirmed that intradental HTMRs that respond to tooth cutting largely represent *Scn10a*+ neurons (22/27, ∼81%, *n* = 4 mice, Figure 3E). We found that cutting and friction produced equivalent, minimal warming of the inner tooth (<3°C, Figures S3I–S3K) that is unlikely to trigger any response and cannot account for our observed differences between response magnitude. These results demonstrate that intradental HTMRs detect and encode the degree of superficial frictional damage.

### Damage responses of intradental HTMRs are independent of Piezo2 or Scn10a (Na_v_1.8)

Our previous work indicated that intradental neurons express the mechanosensitive ion channel *Piezo2*^22^ that underlies mechanosensation in the skin.^10,19,27^ Is Piezo2 required for intradental HTMR activation by enamel cutting? To investigate this, we examined neuron responses after AAV-mediated conditional knockout of *Piezo2* (*Piezo2*-cKO), which circumvents lethality of global *Piezo2* deletion.^10,19^ We first validated loss of *Piezo2* in sensory neurons using ISH against *Piezo2* and *in vivo* calcium imaging. Indeed, we found that neurons that were transduced with AAV-Cre (i.e., GCaMP6f+ neurons) had marked loss of cytoplasmic *Piezo2* using ISH (Figures S3L and S3M). Furthermore, as previously demonstrated,^10,19^ we found that *Piezo2*-dependent innocuous cheek brush largely failed to evoke responses from GCaMP6f+ neurons in calcium imaging in *Piezo2*-cKO mice (Figure S3N). Next, we examined responses of intradental neurons to cutting of the molar tooth in the context of *Piezo2*-cKO. Despite loss of *Piezo2*, we observed no significant change in the proportion of intradental HTMRs responding to cutting (Figures 3F and 3H). These findings indicate that neuronal responses to enamel damage persist despite the absence of *Piezo2* in intradental neurons.

Intradental HTMRs are marked by *Scn10a* expression, encoding the pore-forming α subunit of Na_v_1.8 that contributes to noxious mechanical pain.^28^ We next sought to determine whether *Scn10a* is required for intradental HTMR sensory transduction in response to enamel damage. To this end, we examined neuronal responses in the well-established knockin/knockout *Scn10a*-Cre driver strain^29,30^ after validating genotypes by PCR. As expected, *Scn10a*-Cre enabled capture of intradental HTMRs with similar proportion of cutting responders (Figures 3C, S3A–S3C, and S3O–S3Q). However, in homozygotes where *Scn10a* translation is lost from Cre expression, we found no reduction in the proportion of intradental HTMRs responding to enamel cutting (Figures 3G and 3H). These experiments indicate that the *Scn10a* gene product (α-Na_v_1.8) is dispensable for the transduction of damage responses. Taken together, these experiments suggest that an unknown high-threshold mechanical receptor may underlie damage responses of intradental HTMRs.

### Chemogenetic activation of intradental HTMRs elicits a marked pain phenotype

**Figure 4.**
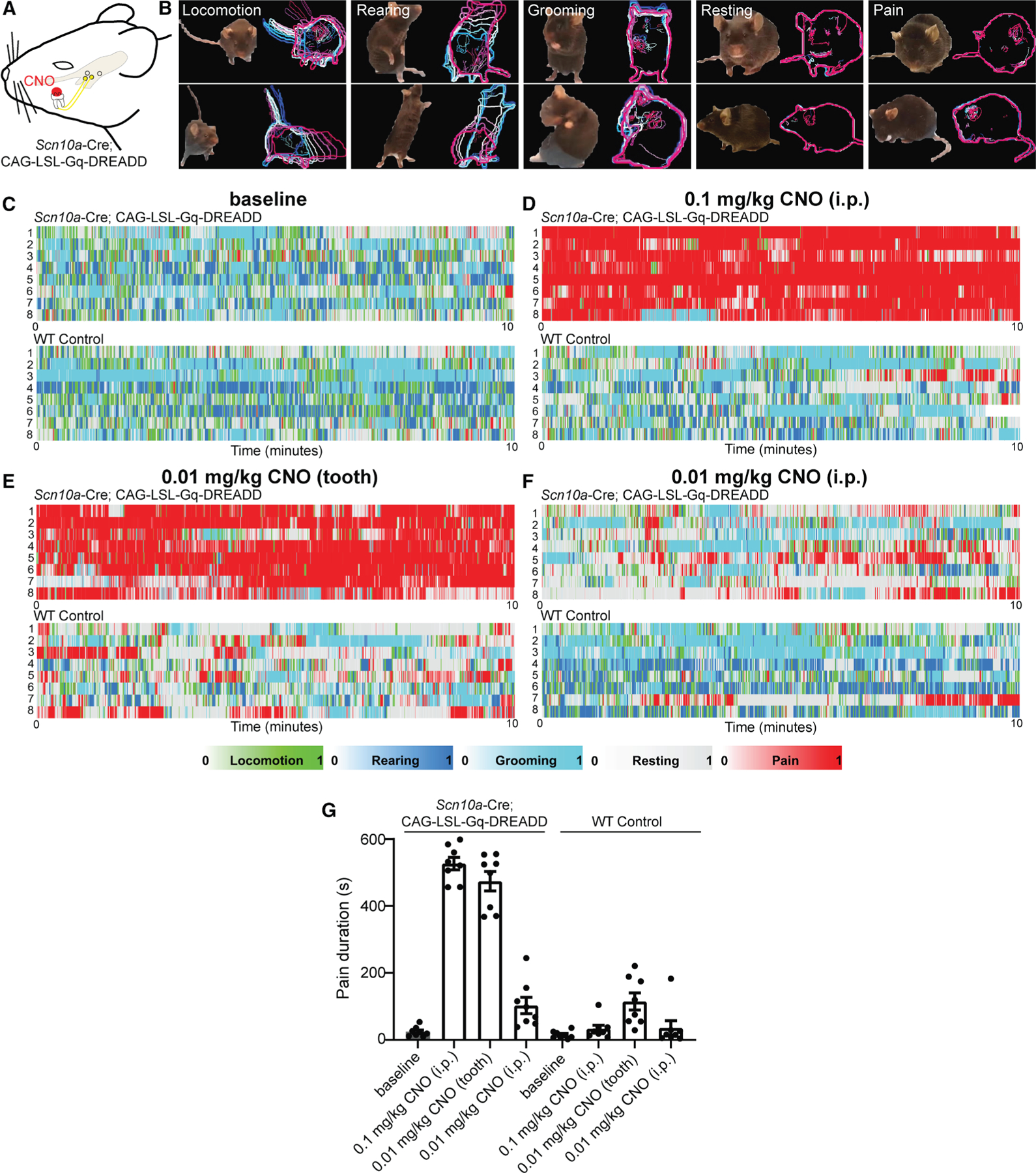
Chemogenetic activation of intradental HTMRs elicits a marked pain phenotype (A) Schematic demonstrating the experimental approach. The excitatory Gq receptor hM3Dq (CAG-LSL-Gq-DREADD) was driven by *Scn10a*-Cre and CNO was directly applied to the tooth. (B) Representative examples showing video frame (left) and corresponding motion pattern images from LabGym (right) to illustrate behavioral categories. Example from the front (top) or side (bottom) for each behavior. Overlaid color curves represent successive time points indicating behavior dynamics. (C–F) Raster plots showing the behavior categorizations over time for *Scn10a-*Cre; CAG-LSL-Gq-Dreadd (top) and wild-type controls (bottom) during (C) baseline, (D) 0.1 mg/kg CNO i.p., (E) 0.01 mg/kg CNO applied to unilateral m1 and m2 mandibular molars, and (F) 0.01 mg/kg CNO i.p. Color bars indicate behavioral categories, with color intensity reflecting the probability of each behavior. Time (*x* axis, s) and with each row (*y* axis) a single animal. *n* = 8 animals/genotype. (G) Bar graph depicting pain duration corresponding to (C)–(F). Plotted individual data points represent the cumulative pain duration for individual animals. Bar shows the mean, and error bars indicate the SEM (see Table S1 for one-way ANOVA with Bonferroni correction). See also Figure S4, Table S1, Videos S1, and S2.

Activation of sensory neurons within the tooth via exposed dentin elicits pain in humans.^31^ Considering our transcriptional analysis and *in vivo* imaging support that intradental neurons represent nociceptive HTMRs, we next sought to confirm that their activation produces pain in freely moving animals. To this end, we crossed the *Scn10a*-Cre driver into the CAG-LSL-Gq-DREADD (hM3Dq) to enable selective activation of *Scn10a*+ sensory neurons using clozapine-N-oxide (CNO). We reasoned that this chemogenetic strategy (*Scn10a*-DREADDq) would permit both broad activation of *Scn10a*+ sensory neurons following i.p. administration of CNO as well as targeted activation^32^ of intradental neurons after application of CNO directly to the tooth (Figure 4A). First, we captured behaviors of *Scn10a*-DREADDq and controls lacking DREADDq at baseline and following administration of CNO (0.1 mg/kg, i.p.). *Scn10a*+ sensory neurons primarily represent nociceptors^9^ and, as expected, their widespread activation in *Scn10a*-DREADDq mice elicited marked postural and orofacial expressions consistent with hunching and grimace that are associated with pain.^33^ These elements were largely absent in baseline or controls even when animals were not moving (i.e., resting). Conversely, behaviors such as locomotion, rearing, and grooming were common in baseline and control videos. Examples of pain, resting, locomotion, rearing, and grooming were extracted from these initial recordings (Figure 4B) and used to train the AI-driven behavioral analysis tool LabGym^34,35^ to enable automated scoring of these behaviors in full-length videos. Quantification confirmed that after 0.1 mg/kg CNO i.p., *Scn10a*-DREADDq mice predominately exhibited pain (Figures 4C, 4D, and 4G; Video S2), while wild-type control mice demonstrated resting, locomotion, and grooming that was comparable with uninjected mice at baseline (Figures S4A–S4E; Video S3). We next sought to evaluate pain following application of CNO to the tooth. While we expect that CNO should remain localized to the site of application, we reduced the dosage of CNO 10-fold to further reduce possible systemic effects of CNO. Remarkably, after 0.01 mg/kg CNO was applied to the tooth (see STAR Methods for details), we found that *Scn10a*-DREADDq mice, but not control mice, exhibited a duration of pain comparable with those receiving 0.1 mg/kg CNO i.p. (Figures 4E and 4G, see Table S1 for statistics). *Scn10a-*DREADDq mice receiving 0.01 mg/kg CNO i.p. exhibited minimal pain (Figures 4F and 4G, see Table S1 for statistics) indicating that pain from CNO applied to the tooth was not due to off-target effects. Furthermore, mice in control groups demonstrated significant increases in locomotion and resting and trended toward more grooming compared with *Scn10a*-DREADDq mice receiving tooth CNO (Figures S4A–S4E, see Table S1 for statistics). Together, these data support that intradental HTMRs are nociceptors that produce pain upon activation.

### Optogenetic activation of intradental HTMRs induces digastric muscle contraction and initiates a protective jaw-opening reflex

**Figure 5.**
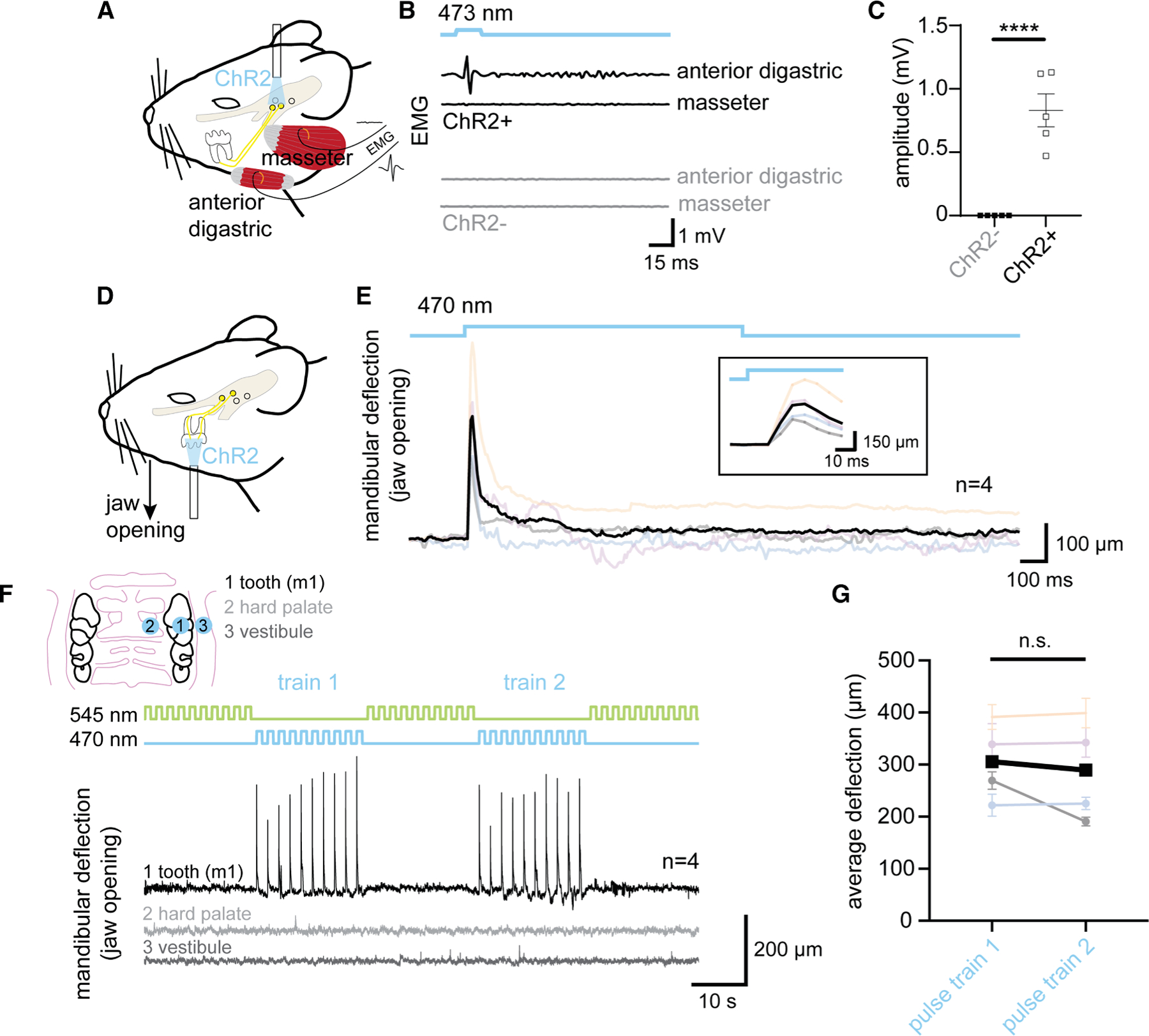
Optogenetic activation of intradental HTMRs induces digastric muscle activity and initiates a jaw-opening reflex (A–C) Optogenetic activation of intradental neurons induces activity in the anterior digastric muscle EMG. (A) Schematic of selective activation of intradental neurons while recording EMG. Channelrhodopsin-2 was selectively expressed in right mandibular intradental neurons innervating m1 and m2 via tooth injection of AAV6/2-hEF1a-iCre into Ai32(RCL-ChR2(H134R)/EYFP) mice. (B) Example traces demonstrating EMG activity elicited by optogenetic activation of intradental neurons (15 ms, 473 nm), *n* = 5 mice for all conditions. Vertical scale bar, 1 mV. Horizontal scale bar, 15 ms. (C) Graph depicting the amplitude of activity of digastric muscle EMG in response to blue light stimulation (15 ms, 473 nm). Plotted individual data points represent the peak-to-peak amplitude of the elicited EMG trace. Error bar indicates SD. *n* = 5 mice per condition. *p* < 0.0001 using unpaired t test. (D–G) Optogenetic activation of intradental neuron terminals in the intact tooth results in mandibular deflection (i.e., jaw opening). (D) Schematic of approach. Channelrhodopsin-2 expression (Ai32) was driven by *Scn10a-*Cre and an optical fiber (500 μm) delivered blue light to target tissue. (E) Traces of average measured mandibular deflection in response to single blue light pulse (470 nm, 1 s) directed to the molar tooth. Black trace represents the average (*n* = 4 mice). Multicolored traces represent average measurements (*n* = 10 traces/mouse). Vertical scale bar, 100 μm. Horizontal scale bar, 100 ms. Inset shows brief timescale of trace from (E). Vertical scale bar, 150 μm. Horizontal scale bar, 10 ms. (F) Average trace of measured mandibular deflection in response to trains of blue or green light pulses (470 or 545 nm, 10 pulses, 0.5 Hz, 1 s). Vertical scale bar, 200 μm. Horizontal scale bar, 10 s. *n* = 4 mice. (G) Line graph depicting average mandibular deflection in response to sequential blue light pulse train stimulation of molar. Average deflection from each mouse is plotted as multicolored individual points and represents mean peak amplitude and error bars show SEM of mandibular deflection during stimulation. *n* = 10 measurements/mouse/train related to traces (F). Black points are average of *n* = 4 mice. n.s. *p* > 0.05 using unpaired t test. See also Figure S5 and Video S4.

In addition to producing pain, somatosensory HTMRs are also known to initiate reflexes to protect structures from damage.^3^ Considering that teeth are at risk of damage from masticatory forces and/or hard food substrates during regular function, we next sought to determine whether activation of the intradental HTMRs might drive protective responses that contribute to fitness. Interestingly, low-intensity electrical stimulation of teeth in larger mammals has been shown to elicit electromyogram (EMG) activity in some muscles of mastication.^36^ We next sought to determine if selective optogenetic activation of intradental neurons was capable of inducing EMG activity in the anterior digastric muscle, which contributes to jaw opening (i.e., mandibular deflection). To this end, we relied on AAV-Cre transduction of intradental neurons via cavitations in the molar teeth to initiate the expression of channelrhodopsin-2 (ChR2) in a Cre-dependent effector strain (Ai32, Figures 5A and S5A). ISH confirmed that AAV transduction targeted intradental neurons since ChR2-YFP+ sensory neurons largely expressed both *S100b* and *Scn10a*+ (103/123 neurons, *n* = 4 mice, Figures S5B and S5C). Activation of intradental neurons expressing ChR2 using blue light stimulation (15 ms pulse) of the TG produced an immediate, transient waveform in the ipsilateral anterior digastric EMG (Figures 5B, 5C, S5D, and S5F–S5H; 0.83 ± 0.13 mV amplitude, *n* = 5 mice). We also found that electrical stimulation of the inferior alveolar nerve elicited comparable EMG activity in the anterior digastric muscle (Figure S5C). Alternatively, we found no EMG activity in response to blue light stimulation when intradental neurons lacked ChR2. The ipsilateral masseter muscle that contributes to closing the jaw also failed to demonstrate EMG activity after light stimulation in either condition (Figures 5B and S5E). Prolonged blue light stimulation (100 ms pulse) elicited similar waveforms in ipsilateral digastric EMG (Figures S5E–S5H; *p* > 0.05), likely reflecting equivalent ChR2 inactivation. Changes in EMG activity were not observed at light offset (Figures 5B, S5D, and S5E). Taken together, these data indicate that somatic optogenetic activation of intradental neurons leads to electrical activity of the anterior digastric muscle.

The anterior digastric muscles function to open the jaw by depressing the mandible.^37^ To directly determine if collective activation of intradental HTMRs initiates overt jaw movement, we next employed a genetic strategy to drive ChR2 expression in these cells using *Scn10a*-Cre. For these experiments, we selectively activated intradental HTMRs by directing the optical fiber onto a single intact molar tooth (Figure 5D). Here, we found that pulses of blue light (470 nm, 1 s) elicited a rapid, brief mandibular deflection (10 ms onset, reversing after 30 ms, Figure 5E; Video S4). Paralleling EMG activity, light offset had no effect on jaw position. We determined that repeated blue light pulses (10 pulses, 0.5 Hz, 1 s) induced a series of mandibular deflections entrained to light onset (Figure 5F) that did not diminish between pulse trains (Figures 5F–5G and S5I) (average deflection 305 ± 37 vs. 289 ± 49 μm, *p* > 0.05). Conversely, green light (545 nm) failed to produce any jaw movements despite identical stimulating frequency and duration (10 pulses, 0.5 Hz, 1 s). Importantly, blue light stimulation of molar-adjacent tissues (i.e., hard palate or oral vestibule) also failed to produce observable jaw movements (Figure 5F). Notably, despite the short latency of jaw movement following blue light stimulation, we found an absence of direct *Scn10a*+ projections to the motor nucleus (5N) controlling the anterior digastric (5ADi) (*Scn10a*-Cre; LSL-ChR2-YFP, Figures S5J and S5K). Taken together, these experiments reveal that optogenetic activation of intradental HTMRs indirectly drives a rapid jaw-opening reflex.

## DISCUSSION

Mammalian teeth are specialized internal organs that endure a range of mechanosensory stimuli during biting and mastication. While intradental neurons have been known to evoke pain through their activation, a biological role had not been defined for this interoceptive innervation using modern neuroscience approaches. Acute pain has clear utility in certain forms of injury. For instance, a fractured bone in the forelimb elicits pain that promotes guarding behavior, reduced use, and healing. However, tooth fracture and pulpal damage may be accompanied by pain, but the pain does not promote healing. Here, we establish an *in vivo* functional imaging approach to identify intradental neurons in an intact tooth, define their responses to mechanical damage of the tooth, and determine that their targeted activation leads to pain and jaw opening. Our experiments reveal a population of intradental HTMRs that (1) detect forces applied to the exposed dentin as well as damage of the superficial enamel, (2) do not contribute to the detection of innocuous forces encountered by the tooth, (3) target the inner dentin with intermingled terminals providing anatomical evidence supporting their coordinated response to stimuli, (4) rely on a Piezo2- and Scn10a-independent mechanism to transduce damage responses, (5) drive pain in a free-moving animal, and (6) lead to digastric muscle contraction and a jaw-opening reflex.

Protective reflexes have been described for the knee (patellar reflex), cornea (eye-blink reflex) and the jaw (jaw-jerk reflex) that occur within 100 ms latencies. Here, we describe an extremely rapid reflex (5–15 ms) that is initiated following the activation of terminals of intradental HTMRs within the dentin and indirectly triggers digastric muscle contraction. We predict that the intradental HTMRs respond when the jaw is closing and one or more teeth make unexpected contact with hard foods (i.e., cartilage or bone) or opposing teeth. In this scenario, the activation of intradental HTMRs would result in the contraction of the digastric, which would ostensibly slow, stop, or reverse the closing mandibular movement. Based on our data, activation of intradental HTMRs within a single tooth during mastication would be sufficient to drive a reflex response. Future work will be required to determine the brainstem circuit that underlies the jaw-opening reflex.

Recent work indicates that *Calca*+ circumferential HTMRs innervating hair follicles in skin mediate a nocifensive response.^3^ Intradental HTMRs are most similar to the CGRP-ζ based on their expression of *Chrna7*.^24^ However, intradental neurons are likely to be distinct from CGRP-ζ neurons found within the DRG considering their lack of *Smr2* and their unique terminal morphology.^17^ Thus, we propose that intradental HTMRs represent a distinct subclass of HTMRs that innervates and protects other vulnerable tissue structures by engaging withdrawal reflexes.

Intradental HTMRs efficiently respond to damage of the tooth indicating they may be tuned for its detection; however, the precise component of the damage stimulus that triggers intradental HTMR responses is not known. The hydrodynamic theory posits that stimuli of varying etiology (e.g., thermal, mechanical, or chemical) converge on microfluidic fluid flow through dentinal tubules, which mechanically activates receptors and triggers sensation.^38^ We cannot rule out that superficial damage of the tooth may contact a portion of dentin on the mouse molar to initiate fluid flow. Our observations that intradental HTMRs respond to cold and direct force following dentin exposure further support this possibility. While ‘‘HTMRs’’ reflects harmonized somatosensory nomenclature,^24^ designating them as ‘‘myelinated mechano-nociceptors’’ would be more accurate if miniscule fluid movements and associated low forces underlie the activation of intradental neurons. Follow-up studies are necessary to provide important distinction. For instance, comparing micro-structural changes in the tooth caused by vibration vs. cutting could reveal the activating component and whether there is accompanying fluid flow. Alternatively, performing *in vivo* imaging in conjunction with precise thermal manipulation of the teeth may enable differentiation of molecular vs. physical gating and uncoupling the dependence of thermal responses on fluid flow.

Intradental neurons have been previously suggested to function as LTMRs.^39^ On the contrary, our data directly show that intradental neurons fail to respond to direct forces or vibration applied to the intact tooth. Instead, it is highly probable that light forces applied to the teeth are transduced by LTMRs innervating surrounding periodontal (i.e., tooth surrounding) tissues. Future studies investigating the molecular identity and innervation targets within surrounding periodontium tissues will shed insight into the mechanisms for how direct mechanical forces applied to the tooth are detected.

We determined that damage responses by intradental neurons are not abolished by their loss of the canonical mechanoreceptor Piezo2 or the nociceptive transmitter Na_v_1.8. We cannot rule out that these membrane channels may contribute to tooth sensation; however, our data suggest that blocking either ion channel in intradental neurons is unlikely to prevent their activation while the teeth are structurally damaged. This is in contrast to a recent clinical trial that supports the effectiveness of inhibition of Na_v_1.8 to diminish post-surgical inflammatory pain.^40^ While we acknowledge that these data represent negative findings, if substantiated, they hint at the existence of unidentified molecular sensors for mechanical stimulation of the tooth. Single-cell sequencing of intradental neurons^41^ toward screening and identification of novel mechanoreceptors and nociceptive transducers will provide additional targets for next-generation dental anesthetics that can be validated with our approach.

Our work provides fundamental insight into the basis and role of tooth sensation, but some open questions remain. While some evidence suggests that the tooth pulp receives innervation from C-fiber nociceptors that contribute to forms of tooth-related pain, our work to date suggests these are unlikely to contribute to the protective reflex response. Our findings do not exclude the possibility that C-fibers may be present deep in the tooth pulp where they could contribute to differential aspects of tooth physiology in the context of inflammation. Furthermore, our study did not explore the contribution of resident pulp cells (i.e., odontoblasts) to dental sensation. While odontoblasts have been proposed as a putative sensory cell based on *in vitro* assays,^42^ these cells lack both vesicles and microscopic proximity to nerve terminals that would enable traditional synaptic transmission. Indeed, neuronal HTMR counterparts in the epidermis skin terminate as free nerve endings^43^ and act as the primary sensory cell. Future experiments will be necessary to determine if activation of odontoblasts elicits responses from intradental neurons. Clinically, we predict that intradental HTMRs may underlie acute pain during biting as part of cracked tooth syndrome.^44^ Further work will be needed to determine if responses of intradental HTMRs are altered by the inflammatory milieu and can explain other forms of dental pain. For instance, intradental HTMRs may exhibit lower threshold responses to mechanical stimulation in the context of tooth infection, and begin to contribute to the exquisite pain associated with toothache.

Our study indicates that intradental HTMRs respond to damage, initiate an important protective reflex, and drive overt pain behaviors. Chemogenetic activation of intradental HTMRs represents a model for orofacial pain that can be leveraged to evaluate analgesics and reveal orofacial features that correlate with the experience of pain. Further experiments in awake animals would examine if the jaw-opening reflex and pain from the activation of intradental neurons are necessarily concurrent.

### Limitations of the study

We provide direct evidence that most intradental neurons, as captured by CTB+ retrograde labeling, express *Chrna7*. While intradental neurons captured by electrical stimulation primarily represent HTMRs (*S100b*+, *Scn10a*+), we did not validate that neurons captured by functional imaging also represent the subset of *Chrna7*+ HTMRs. Experiments that utilize Cre driver lines that differentiate subtypes of HTMRs would be compatible with *in vivo* imaging and chemo-/optogenetic experiments and provide further molecular definition of the intradental neurons.

We conducted key gain-of-function experiments providing insight into the function of intradental neurons. First, chemogenetic activation of *Scn10a*+ neurons by application of CNO to the tooth elicits pain behaviors. Second, optogenetic activation of *Scn10a*+ neurons via placement of an optical fiber on the tooth elicits a jaw-opening reflex. Last, complementary experiments demonstrated that AAV transduction via the tooth captures *S100b*+, *Scn10a*+ intradental neurons and direct activation of these somas elicits EMG activity in the digastric muscle. These experiments alone cannot indicate that intradental neurons are sufficient in driving these outputs. Furthermore, while *Scn10a* is almost exclusively expressed in somatosensory neurons (and nociceptors), we have not demonstrated direct cellular activation of *Scn10a*+ intradental neurons by either chemo-/optogenetic methods.

## RESOURCE AVAILABILITY

### Lead contact

Further information and requests for resources and reagents should be directed to and will be fulfilled by the lead contact, Dr. Joshua J. Emrick, DDS, PhD (jjemrick@umich.edu).

### Materials availability

This study did not generate new unique reagents.

### Data and code availability

- All original code has been deposited at Zenodo at https://doi.org/10.5281/zenodo.15643732 and is publicly available as of the date of publication.
- All DeepLabCut or LabGym models or microscopy data will be shared by the lead contact upon request.
- Any additional information required to reanalyze the data reported in this paper is available from the lead contact upon request.

## STAR★METHODS

### EXPERIMENTAL MODEL AND STUDY PARTICIPANT DETAILS

#### Animal assurance statement

All animal experiments were performed in accordance with protocols approved by the University of Michigan Institutional Animal Care and Use Committee following NIH guidelines. Experiments were performed with male and female mice. Number of mice used are indicated in the figure legends for each experiment. Mice were group housed at room temperature with *ad libitum* access to standard lab mouse pellet food and water on a 12 hr light/12 hr dark cycle. Mouse lines were purchased from Jackson or received from listed contributors, and crossed with reporter lines without additional backcrossing.

#### Mouse lines

The mouse lines used in the present study included Ai95(RCL-GCaMP6f)-D (C57BL/6J) (#028865, JAX), Piezo2^lox/lox^; Tac1-tagRFP-2a-TVA line,^10^ *Snap25*-LSL-2A-EGFP-D (#021879, JAX), Nav1.8-Cre (#036564, JAX), CAG-LSL-Gq-DREADD (#026220, JAX), C57BL/6J (#000664, JAX) and Ai65(RCF-tdT), Ai32(RCL-ChR2(H134R)/EYFP) (#024109, JAX). *S100b*-Cre is an unpublished mouse line from Dr. Nicholas Ryba that was validated in this study. For the main body of this study, Ai95D transgenic mice were bred and GCaMP6f expression was induced via postnatal AAV-Cre injection.

### METHOD DETAILS

#### AAV Viral Delivery

To broadly induce GCaMP6f expression in trigeminal ganglia (TG) neurons in Ai95D and *Piezo2*-cKO transgenic mice, postnatal day 0–3 mouse pups were injected intraperitoneally with AAV9-Cre. Prior to injection, mouse pups were transiently anesthetized by placing pups on a plastic Petri dish on ice. A Hamilton syringe (Hamilton Company, Reno, USA) was used to inject 10^12^ viral genomes in a 10 μL volume of ssAAV-9/2-hEF1a-iCre-WPRE-bGHp(A) (Physical titer: 8.1 × 10E12 vg/mL, catalog #v225–9, University of Zurich Viral Vector Facility VVF, Zurich, CH) diluted in sterile saline. To induce GCaMP6f expression while simultaneously co-expressing mCherry in the TG of Ai95D mice for post hoc ISH alignment following calcium imaging, Ai95D mice were postnatally injected via the same protocol with ssAAV-9/2-hSyn1-chI-mCherry_2A_iCre-WPRE-SV40p(A) (Physical titer: 5.6 × 10E12 vg/mL, catalog #v147–9, University of Zurich Viral Vector Facility VVF, Zurich, CH). To validate transduction efficiency of Ai95D mice injected with AAV-Cre at P0, TG sections underwent ISH for *GCaMP* and *Tubb3*. In ImageJ, ROIs for individual neurons were drawn based on *Tubb3* expression, followed by manual scoring for the presence of *GCaMP*, with *GCaMP*+ positive cells defined based on visible presence of fluorescent signal above background throughout the ROI. Transduction efficiency was calculated as the percent of GCaMP+ cells/*Tubb3*. Somal diameter for GCaMP+ versus GCaMP-cells was also calculated using Feret’s diameter for each ROI (ImageJ). To validate knockout efficiency in *Piezo2* cKO mice, TG sections from Piezo2-cKO; Ai95D or control Ai95D mice injected with AAV-Cre at P0 underwent ISH for *GCaMP*, *Piezo2,* and *Tubb3*. Individual neurons from imaged sections were analyzed for the presence of *GCaMP* and *Piezo2*. In ImageJ, ROIs for individual neurons were drawn based on *Tubb3* expression, followed by manual scoring for the presence of *GCaMP*, with *GCaMP*+ positive cells (indicating Cre recombination) defined based on visible presence of fluorescent signal above background throughout the ROI. Next, ROIs taken from both *Piezo2-cKO* and control Ai95D mice were blindly shuffled using a custom MATLAB application and manually evaluated for *Piezo2* expression. ROIs were scored as positive for *Piezo2* based on the presence of diffuse fluorescent puncta throughout the cytosol.

Sparse labeling of intradental neurons in Cre lines was achieved via two AAV injection approaches. A cohort of adult (6–8 week old) *S100b*-Cre mice were injected with AAV9-CAG-FLEX-tdT (Physical titer: 1–9 × 10E13 vg/mL, Vigene) bilaterally to the trigeminal ganglion by passing through the medial wall of the orbit. Virus was diluted 1:1 in 1X sterile PBS and 1.3 μL injections were delivered to each TG. Mice were anesthetized using the SomnoSuite Low-Flow Anesthesia System (5% induction, 2.5% maintenance, Kent Scientific). Two weeks following injections, mice were euthanized, perfused, and TG and jaws were dissected and processed as described below. Sparse labeling of *S100b*+ neurons relied on variable viral transduction efficiency. For sparse labeling of intradental neurons in *Scn10a*-Cre mice, we performed neonatal AAV injections using a Brainbow viral transduction approach. Using the protocol described above for neonatal injections, pups were injected with a 10 μL volume of a 1:5 dilution of pAAV-EF1α-Brainbow-invert tagBFP-eYFP-WPRE (Physical titer: 2.2 × 10E13 vg/mL, catalog #V120025, Addgene) and pAAV-Ef1a-Brainbow/mCherry/mTFP-WPRE (Physical titer: 2.2 × 10E13 vg/mL, catalog #V160749, Addgene) diluted in sterile saline. At 8 weeks of age, animals were euthanized, perfused, and TG and jaws were dissected and processed as described below. It is worth noting we observed robust expression of all four Brain-bow fluorophores (BFP/YFP/mCherry/TFP) in TG; however, labeling of intradental terminal endings was limited to YFP detected using IHC. We suspect this may be due to deficiencies in terminal ending trafficking of the three other fluorophores.

To selectively induce channelrhodopsin-2/EYFP fusion protein in intradental neurons, adult (8 weeks old) Ai32(RCL-ChR2(H134R)/EYFP) mice were anesthetized and access to the maxillary or mandibular molars was achieved using the surgical protocol described for retrograde labeling below. Shallow occlusal cavitations were prepared on the right maxillary and mandibular first (m1) and second (m2) molars. Hydrophobic bonding agent was applied to surrounding tissues to prevent off target exposure to surrounding tissues. A micropipette tip was used to precisely inject 10^12^ viral genomes to the exposed cavitations using a total volume of 2 μL of 1:1 5% silk fibroin (Advanced BioMatrix, Cat. No. 5154–20ML): ssAAV-6(F129L)/2-hEF1a-iCre-WPRE-bGHp(A) (Physical titer: 7.2 × 10E12 vg/mL, catalog #225–6(F129L)/2, University of Zurich Viral Vector Facility VVF, Zurich, CH). The total volume was delivered using 3 applications over 10 min allowing each application to gently dry then the preparation was covered with a dental filling (Flow-It ALC B2; Pentron) then light cured. Mice were allowed to recover for at least 3 weeks to ensure adequate viral-induced expression prior to optical fiber implantation (described below).

#### Retrograde labeling of intradental neurons

Retrograde labeling of intradental trigeminal neurons via cholera toxin B-subunit (CTB) reconstituted in Milli-Q water was performed as described previously.^12^ Briefly, adult mice (over 6 weeks of age) were deeply anesthetized via isoflurane administration (4% for induction and 1.5–2% for maintenance using a SomnoSuite Low-Flow Anesthesia System (Kent Scientific) administered through a secured nose cone. Ophthalmic ointment (Fisher Scientific, Catalog #NC0490117) was applied to the eyes to prevent drying. Body temperature was maintained using a hand warmer. Access to the mandibular molars was achieved via a custom device designed to separate the maxillary and mandibular incisors to open the mouth vertically, followed by oral insertion of surgical retractors horizontally to expose the molars. Shallow occlusal cavitations were prepared with a ¼ round carbide dental bur attached to a micromotor drill (variable RPM). Hydrophobic bonding agent was applied to surrounding tissues to prevent nonspecific labeling. CTB volume (∼1 μL per tooth) was delivered using 3 applications over 15 min allowing each application to gently dry then the preparation was covered with a dental filling (Flow-It ALC B2; Pentron) then light cured. Given our previous report that 16 hr is sufficient to induce robust CTB labeling of intradental neurons, mice were allowed to recover for this amount of time prior to either harvesting TGs for ISH or conducting *in vivo* imaging experiments. For *in vivo* imaging, tooth fillings were removed immediately prior to the experiment.

#### Immunohistochemistry

Animals were perfused using 30 mL of 4°C 1X PBS followed by 4% PFA/1X PBS. TG were dissected and fixed in 4% PFA for 2 hr then incubated overnight in 30% sucrose/1X PBS at 4°C until they had sunk, after which they were placed in OCT, frozen with dry ice, and stored in −80°C until sectioning. For evaluation of immune cell in response to dentin or pulp exposure surgeries were performed to expose the dentin or pulp (as described below in tooth stimulation) using the same time course as with calcium imaging experiments before perfusions. Mandibles and maxillae were dissected and post-fixed overnight in 1X PBS, 4% PFA. Samples were then demineralized for 7 days in 0.1 M EDTA at 42°C shaking (350 RPM) with buffer exchange every 2 days. After demineralization, the jaws were cryoprotected in 30% sucrose/1X PBS for 1–2 days until they had sunk, after which they were placed in OCT, frozen with dry ice, and stored in −80°C until sectioning. OCT embedded tissues were sectioned (Leica CM1950) at 20 μm (TG) or 30 μm (hemisected whole skulls) and thaw captured onto Gelatin Subbed Slides (Southern Biotech, #SLD01-BX). Slides were stored in −80°C until immunohistochemistry was performed. Slides were brought to room temperature then a hydrophobic barrier was drawn around the tissues using an ImmEdge Pen (Vectorlabs). Slides were post-fixed using 1X PBS/4% PFA for 10 min then washed with 1X PBS three times. Sections were blocked with 10% Normal Goat or Donkey Serum in 0.1% Triton X-100/1X PBS for 1 hr at RT, then incubated in primary antibody 4°C overnight. After washing 3 times in 1X PBS, sections were then incubated in secondary antibody for 2 hr at RT in dark. Finally, sections were washed 3 times in 0.1% Triton X-100/1X PBS then mounted in Prolong Diamond with or without DAPI (Life Technologies) coverslipped, then imaged using a confocal microscope (Olympus FV3000, Evident Scientific, Inc.).

##### Quantification of immune cell fluorescent intensity

Fluorescence quantification of immune cells was performed using a MATLAB-based image processing workflow. Images were first pre-processed by excluding any bright areas outside the region of interest—the pulp and surrounding dentin of the tooth. The DAPI channel, due to its brightness and clear delineation, was used to define ROIs. Thresholding was applied to identify these regions, followed by morphological operations to clean up noise by removing small objects and filling holes. Once ROIs were identified, the immune cell fluorescence signal was normalized by subtracting the mean intensity of non-ROI background areas, ensuring consistent signal comparisons across images. For enhanced accuracy, background fluorescence was subtracted. This involved thresholding the background channel to identify significant areas of noise and subtracting these from the immune cell channel. Fluorescence intensity was quantified by calculating the average brightness within the ROIs, achieved by dividing the total fluorescence intensity of the ROIs by their total area. This approach enabled precise quantification while accounting for variations in background intensity and ensuring consistency across different images. Visual checks were implemented throughout the process to ensure accuracy in thresholding.

#### *In situ* hybridization (ISH) of TG in tissue sections

Mouse TG were freshly dissected, embedded in OCT, and flash frozen on dry ice before cryosectioning (Leica CM1950) at a thickness of 20 μm onto Superfrost Plus Slides (Fisher #12-550-15). Slides were then stored in −80°C until use. *In situ* hybridization (ISH) was performed via either a modified hybridization chain reaction (HCR) version 3 protocol^3,10,12^ or a modified RNAscope protocol.

For the HCR method, buffers, hairpin amplifiers and probes against transcripts for mouse genes *S100b, Calca, Scn10a, Mrgprd, Chrna7, Fxyd2*, *mCherry*, *TdTomato, eYFP*, *Piezo2*, or *Tubb3* were purchased from Molecular Instruments. Slides were fixed in 4% PFA/1X PBS for 15 min on ice, then washed 3X with 1X PBS. Following fixation, sections were acetylated with 0.3% v/v acetic anhydride and 0.1 M triethanolamine in Milli-Q water and washed 3X in 1X PBS before undergoing dehydration through an ethanol series (50%, 70%, 100%, fresh 100%; 5 min each) then rinsed 3X in 2X saline sodium citrate (SSC). Next, slides were prehybridized with hybridization buffer using a Coverwell chamber (Grace Biolabs) at 37°C for 10 min in a humidified hybridization oven. This was followed by incubation with a working hybridization solution (prepared by adding probes with adapters B1-B6 at a concentration of 4 nM per probe to pre-warmed hybridization buffer at 37°C), and incubated for 1–3 days in a humidified hybridization oven at 37°C. Following hybridization, slides were washed in 100% wash buffer for 5 min at 37°C then underwent a wash solution series (75% wash buffer in 5x SSC containing 0.1% Tween 20 (SSCT), 50% wash buffer in 5x SSCT, 25% wash buffer in 5x SSCT, 100% 5x SSCT for 10 min at 37°C) with a final wash in 100% 5x SSCT for 5 min at room temperature (RT). Next, slides were pre-incubated in amplification buffer for 30 min followed by overnight incubation in a working amplification solution, at RT in a humidified chamber protected from light. The working amplification solution consisting of amplification buffer containing amplifier hairpins for adapters B1-B6 conjugated to fluorophores was freshly prepared according to manufacturer recommendations, ensuring the selected associated fluorophores (Alexa 488, Alexa 561, Alexa 594, Alexa 647, Alexa 750) had no overlap with any endogenous fluorescence present in the sample for each experiment. Finally, slides were washed 2x in 5X SSCT for 15 min with gentle agitation then mounted in Imaging Buffer (3 U/mL pyranose oxidase, 0.8% D-glucose, 2X SSC, 10 mM Tric HCl pH 7.4, 400 U RNAse inhibitor), coverslipped, and sealed for imaging using Cytobond (Scigene).

For RNAscope of retrograde labeled TG, all reagents and probes against transcripts for mouse genes *S100b*, *Smr2,* or *Bmpr1b* were purchased from ACD Bio. RNAscope was performed using published protocols with slight modifications while following ACD Bio manufacturer guidelines. Briefly, slides were removed from −80°C storage and fixed for 1 h with freshly made 4% PFA in 1X PBS precooled to 4°C on ice. Next, slides were rinsed twice with 1X PBS to remove excess fixative, then covered in 1X PBS, coverslipped, and imaged using confocal microscopy for retrograde labeling of CTB-AF647. We chose to screen for CTB at this time as we noticed considerable loss of CTB-AF647 signal following later Protease IV treatment. Tissue sections were protected from damage using spacers created from strips of electrical tape (200 μm thick) that were adhered to the perimeter of coverslips prior to mounting. Following confocal imaging for CTB, coverslips were gently removed and slides were briefly rinsed in 1X PBS before undergoing dehydration through an ethanol series (50%, 70%, 100%, fresh 100%; 5 min each). After dehydration, slides were removed from 100% ethanol and allowed to air dry for 5 min at RT. A hydrophobic barrier was drawn around each section using an ImmEdge hydrophobic barrier pen (Vectorlabs). Slides were then rehydrated in 2X SSC and washed 2X in 2X SSC, then incubated with Protease IV for 10 min, followed by two washes in 2X SSC. Next, slides were incubated in a mix of RNAscope probes for 2 hr in a humidified hybridization oven at 40°C. Slides were then washed 2X in Wash Buffer for 2 min each at RT, followed by adding and washing Amp1, Amp2, and Amp3 then developing HRP C1 and C2 signals according to manufacturer instructions. Finally, slides were mounted using Prolong Gold Antifade Mountant with DAPI (Thermofisher, P36935), coverslipped, and imaged using a confocal microscope (Olympus FV3000, Evident Scientific, Inc.). Image alignment was performed using ImageJ prior to scoring for CTB and ISH overlap. To quantify gene expression within intradental neurons labeled either via CTB retrograde tracing or AAV-Cre tooth injections in tissue sections following HCR or RNAscope, ROIs were first drawn around labeled intradental neurons. Expression of each probe was manually scored as positive based on visible presence of fluorescent signal above background throughout the ROI.

##### Specificity/efficiency calculation

Cell segmentation in microscopy images of the trigeminal ganglion was performed with the aid of Cellpose, a neural network for biological segmentation tasks. The *cyto* pre-trained model was tuned to our dataset using the Cellpose human-in-the-loop functionality for significant gains in segmentation accuracy. Cellpose masks were manually filtered and adjusted to remove any background artifacts or to fix segmentation borders. The masks were then visually inspected across fluorescence channels before calculating labeling efficiency and specificity. The efficiency metric reflects the proportion of labeled neurons within an imaging field, while the specificity metric reflects a fluorophore’s fidelity to a molecular marker.

#### *In vivo* epifluorescence Ca^2+^ imaging of the trigeminal ganglion

*In vivo* Ca^2+^ imaging of trigeminal neurons was performed in anesthetized mice using previously described methods with slight modifications.^3^ Briefly, mice were deeply anesthetized via isoflurane administration (4% for induction and 1.5–2% for maintenance using a SomnoSuite Low-Flow Anesthesia System (Kent Scientific) administered through a secured nose cone. Ophthalmic ointment (Fisher Scientific Catalog #NC0490117) was applied to the eyes to prevent drying. Body temperature was maintained using a hand warmer. Whiskers were trimmed to ensure that unintentional deflection responses of trigeminal neurons did not occur during imaging. Mice were head-fixed using a custom stereotaxic apparatus that stabilized the skull while enabling access to the oral cavity. Optical access of the trigeminal ganglion surface was achieved via bilateral hemispherectomy as described previously.^3^ Following trigeminal ganglion exposure and establishment of hemostasis, a spring-loaded wire was placed in between the maxillary and mandibular incisors to open the mouth via applying gentle tension vertically, followed by positioning of insulated retractors horizontally to expose the mandibular molars. Ca^2+^ imaging was performed within 1 h of trigeminal ganglion exposure using a Thorlabs custom-built epifluorescence microscope with a 4x, 0.16 NA Olympus objective. Two Ultra-High-Power LED Controllers (Prizmatix) were used to image GCaMP6f (480 nm) and RFP (561 nm) and were filtered using a GFP filter sets (Thorlabs) and tdTomato filter sets for RFP (Thorlabs). Image acquisition was performed using a pco.panda 4.2 bi scientific CMOS camera at 5 Hz. For all experiments in which no post-hoc *in situ* hybridization was performed, a piezoelectric actuator (NanoScan NPC-D-6111, Prior) was used to achieve highly precise vertical linear movement of the Olympus objective in order to capture a deeper field of view (300 μm) of the surface of the ganglion. To verify Piezo2-cKO in live imaging experiments, gentle stroking of the exterior mouse cheek with the grain of the hairs via a paintbrush was performed similar to previous reports.^3,10,19^ Care was taken to ensure each stroke was consistent in gentle applied force resulting in slight deflection of hair. Cheek pinch was performed using forceps to pinch the external cheek skin.

#### Tooth stimulation

***Electrical stimulation*** was used to identify intradental neurons (see below) using a pulse generator (Koolertron DDS Signal Generator). For typical experiments: An insulated wire cathode was placed in the nearby lingual gingiva (see Figure S1). The tip of an insulated wire anode (+) was held on the molar occlusal surface. Following anode placement, a square waveform set to 2–4 V, 0.6 Hz, 1–2 V offset, and 12% duty pulse wave was delivered to a single molar. The anode was moved to the adjacent molar without changing the cathode position. Following the stimulation of both molars, the cathode position was changed. Anode stimulation proceeded as described. This process was repeated for 3 independent rounds with different cathode positions. Intradental neurons were defined as those cells that responded to at least 2 of 3 rounds of electrical stimulation. Cold application was applied to m1 and/or m2 molars as indicated via application of Hygenic Endo-Ice Pulp Vitality Refrigerant Spray (Coltene) that was sprayed on a small spherical cotton pellet (1 mm diameter) for 5 s then immediately applied. ***Enamel cutting*** was performed by application of a ¼ round carbide dental bur (Munce discovery burs, CJM Engineering) driven by a cordless micromotor drill (35,000 RPM) to the occlusal surface of individual molars in a posterior to anterior sweeping motion. Friction stimulus was delivered using the same approach using a rounded ‘‘fluteless’’ carbide dental bur that was smoothed using sandpaper to generate diminutive damage to the molar occlusal surface. Care was taken to ensure each motion and the downward force was consistent between friction and cutting trials in series in the same mouse. In experiments, a single molar was the target of enamel cutting, but the relative size of the bur to the tooth sometimes resulted in hitting the surface of the adjacent molar. Degree of damage to the enamel occlusal surface depended on whether the bur was standard or fluteless (see Figure S5A). ***Vibration*** was delivered using a custom applicator that held a dental file placed in contact with the surface of a single tooth as previously reported.^10^ Briefly, sinusoidal waveforms at 50, 75, 100, 150, 200 Hz lasting 3 s for each were generated using a Digidata 1550 (Molecular Devices) to control a solenoid (Solid Drive SD1sm, Induction Dynamics) and were amplified by a 70 W subwoofer plate amplifier (SA70, Dayton Audio). ***Direct forces*** were applied to the occlusal surface of individual molars via von Frey filaments (0.4 g and 2 g, Ugo Basile) or a dental file (Dentsply) with an applied force that was measured at 20 g. For pulp/dentin exposure experiments, occlusal cavitations exposing the dentin and/or pulp were made just before or during live imaging depending on the experimental condition. Pulp/dentin exposure was performed based on our previously published protocol for retrograde labeling of intradental neurons^22^ and as described above. We defined pulp exposure when drilling depth resulted in presentation of stereotypical visual cues including pink to red coloration and fluid emanating from the pulp bed. Conversely, dentin drilling was achieved via drilling through the enamel while avoiding exposure depths that gave rise to pulp exposure visual cues. Depth of drilling was carefully assessed for each tooth under high magnification with a stereoscope.

***Pulp temperature*** was measured in separate experiments to determine the internal pulp temperature during enamel cutting and friction. Mice were anesthetized and access to the mandibular molars was achieved using protocols previously described for live imaging. A tunnel was drilled into the mesial side of the m1 molar using a needle carbide dental bur (Dentsply) driven by a cordless micromotor drill operating at 35,000 RPM. Drilling extended into the inner pulp chamber while keeping the occlusal tooth structure intact. A T-Type thermocouple (5SC-TT-T-40–36, Omega Thermocouples) was then implanted into the dental pulp. The thermocouple was sealed with Flow-It ALC B2 (Pentron) and light-cured to secure the preparation. Temperature readings were captured using a temperature reader from Sable Systems International (TC-2000 Type T Thermocouple Meter), capable of measuring temperatures from −100°C to +125°C with a resolution of 0.01°C. Data collected by the thermocouples were converted for computer analysis using a LABTRAX 4-Channel Data Acquisition system from World Precision Instruments (WPI Data Acquisition). Data analysis and recording was performed with LabScribe V4 software (IWORX). The standard temperature recording protocol involved a baseline measurement for 30 s, followed by enamel cutting applied for 20 s (between 30 s and 50 s into recording), and continued recording up to 2 min. This protocol enabled reproducible evaluation of thermal dynamics of the inner tooth and pulp in response to enamel cutting/friction.

#### Calcium imaging analysis

##### Motion correction of in vivo Ca^2+^ response time-lapse image data

Image stacks representing multiple stimulation paradigms from a single imaging trial were first concatenated using ImageJ. Inter-frame movement correction was performed using an in-house MATLAB code. The algorithm initially assumes translation alignment for a rough approximation, followed by a precise affine alignment fine-tuning. Both steps utilize mean squares as the similarity metric and employ regular step gradient descent for optimization.

##### Regions of interest (ROIs) seleztion

Regions of interest (ROIs) were manually drawn using the freehand tool in ImageJ around neurons that fired synchronously with the applied electrical stimulation.

##### Fluoreszenze dynamizs

A previously published MATLAB script^10^ was adopted to calculate GCaMP6f fluorescence (ΔF) and correct for fluctuations in background signals for the manually drawn ROIs. Neuropil region local to each somatic ROI was created to enable spatial averaging of the Ca^2+^ response then subtraction from the somatic average. This process was repeated for every frame and corrects for all contaminant signals (i.e., underlying, out of focus, and neighboring cell fluorescence) throughout the experiment.

##### AUC

Area under the curve measurements were made using GraphPad Prism. In particular, the measurements for AUC were taken as *total* area under the curve. There was no user preference for peak intensity or peak width. Simply, the area under the curve was set to be the area under the normalized intensity values during time of stimulation (frames 40–120).

##### Proportions

Proportions were calculated on GraphPad Prism as a function of the number of responses of all voltage responders. Responders were identified using known time of stimulus application (typically around frame 40).

##### Somal diameter

Intradental neuron somal diameter was calculated using ImageJ’s Measure feature (with Set Measurements - Feret’s diameter selected) for each ROI.

#### Post hoc ISH and alignment and background subtraction

For live imaging experiments followed by post hoc ISH, Ai95D mice injected with AAV-Cre-mCherry (see AAV viral delivery) were used to induce sparse mCherry expression across the dorsal TG surface which were used as guideposts for subsequent image alignment. Immediately following live imaging of the dorsal surface of the TG, an additional single image of the endogenous mCherry expression was taken in the same imaging field as the Ca^2+^ recording. Immediately after, the TG was immediately flushed with PBS within the skull to remove residual blood, followed by brief fixation in ice-cold 4% PFA/1X PBS. TG were then dissected and processed for whole mount ISH using HCR as previously described.^10^ Briefly, ganglia were immersed in 4% PFA/1X PBS on ice for 90 min, rinsed 3 times in 1X PBS, and the ventral side of the TG was adhered to a small plastic strip (10 × 2mm) to aid handling, then rinsed once more in 1X PBS. ISH was performed via a modified protocol using hybridization chain reaction version 3.^3,10^ Pre-hybridization: 30 min, 35°C in HCR hybridization buffer (Molecular Instruments); Hybridization: 48–72 hr 35° combining the following probes: *d2eGFP* (detects GCaMP expression)*, mCherry, S100b, Calca, Scn10a, Mrgprd*. Following hybridization, ganglia were washed: 1) 2 × 15 min in wash buffer (Molecular Instruments) at 39°C, 2) 4 × 30 min in 30% formamide/2x SSC at 39°C 3) 2x in 2x SSC at RT (briefly), 4) 1 × 30 min in amplification buffer (Molecular Instruments) at RT rotating in dark, then amplified overnight rotating in dark in amplification buffer (Molecular Instruments) containing amplifier hairpins for adapters B2 - B6 conjugated to AF750, AF488, AF546, AF594, or AF647 prepared as directed by the manufacturer. Following amplification, ganglia were washed 4 × 30 min in 5x SSC/0.1% Tween rotating in dark, then embedded in 2% low-melt agarose filled halfway in a CoverWell imaging chamber (Grace Biolabs). Once agar solidified, the remainder of the chamber was filled with Imaging Buffer (3 U/mL pyranose oxidase, 0.8% D-glucose, 2x SSC, 10 mM Tris-HCL ph 7.4) containing 10 μL RNase inhibitor (New England Biolabs), and ganglia were imaged immediately using an Olympus FV3000 confocal microscope.

For aligning calcium imaging data with post hoc ISH, two mCherry reference images were taken to be used as guideposts, one prior to calcium imaging (*in vivo* mCherry) and one following ISH (post hoc ISH using a probe for *mCherry*). This method was adopted from a previously established protocol^10^ incorporating custom codes for image alignment (https://doi.org/10.5281/zenodo.15643732). The *in vivo* mCherry image was designated as the fixed source, while the post hoc ISH mCherry image was considered a moving target. 8–10 pairs of control points distributed across the full surface of the TG were manually marked between the two mCherry reference images using a custom-made GUI. These control points were used to generate an initial alignment using a second-order polynomial transformation. This was followed by non-rigid alignment using the Demons algorithm, performed across four resolution levels with decreasing iterations [5,1,1,1]. Prior to all registration steps, images were normalized by scaling their intensity values between the 0.1st and 99.9th percentiles to a 0–1 range. Computed transformations were applied uniformly to the post hoc ISH image stack. Following alignment of calcium imaging data and post hoc ISH, electrically responsive cells identified in the Ca^2+^ imaging data were manually outlined in ImageJ and saved as regions of interest (ROIs). These ROIs were overlaid onto the aligned ISH images to verify accurate registration by comparing cellular morphology.

To determine the molecular expression of electrically responding intradental neurons, post hoc ISH for candidate genes were manually scored as positive based on visible presence of fluorescent signal above background throughout the ROI.

#### Electrical and optogenetic stimulation and EMG measurements

##### Fiber optic and muscle electrode implantation, optogenetic stimulation, and EMG recording

Four weeks post-injection of AAV6/2-hEF1a-iCre into the right-side molars of Ai32(RCL-ChR2(H134R)/EYFP) mice, a fiber optic was implanted on top of the right TG. After preemptive administration with 5mg/kg of analgesic carprofen, mice were anesthetized with isoflurane (4–5% for induction, 1.5% for maintenance) before being secured in the stereotactic frame (David Kopf Instruments). Body temperature was maintained at 35°C–37°C using a feedback-controlled heating pad (Physitemp, TCAT-2LV). The following coordinates were used for implantation of the fiber optic: (TG) +2.4 mm from the midline; −3.6 mm posterior to the bregma, −4.7 mm ventral from the bone surface. The fiber optic (200 μm core, RWD) was affixed to the skull using dental cement (C&B Metabond, Parkell).

For the placement of electromyographic activity (EMG) electrodes, a 4-pin head-mount connected to wire electrodes was affixed to the skull with dental cement. Subsequently, all wire electrodes were positioned under the face skin and placed on the surface of each muscle. Incisions were made in the ventral part of the mandible to expose the right anterior digastric, and the right cheek to expose the masseter muscle, respectively. Two stainless steel wire electrodes (36 AWG size, Cat: 36744MHW, Phoenix wire) were inserted 2 mm apart into each muscle and then sutured with a 7–0 polypropylene surgical suture (Prolene TM, Ethicon). One week later, EMG signals of the anterior digastric and masseter muscle were recorded from awake animals by laser pulse administration (15 or 100 ms, 1 s) via a fiber connected to a 473 nm laser. The EMG signals were recorded using an amplifier (Quad Bio Amp; AD instruments), filtered digitally (band-pass filter at 100–3000 Hz), and integrated (time constant of 0.1 s). All the equipment were connected to PowerLab 8/35 (AD instruments) for data acquisition. Following completion of experiments, animals were euthanized, perfused, and TG and jaws were dissected, screened for ChR2-YFP expression, flash frozen on dry ice in OCT, sectioned into 20 μm slices using a cryostat, and mounted on Superfrost Plus Slides (Fisher #12-550-15) for ISH.

##### IAN stimulation

Stimulating the inferior alveolar nerve (IAN) was performed as previously reported.^45,46^ Adult wildtype mice were anesthetized with urethane (1000 mg/kg), with a second dose (600 mg/kg) after 30 min; supplementary doses (100 mg/kg) were administered if required. EMG electrodes were implanted as described above. The IAN was reached intraorally and a monopolar tungsten electrode (impedance 0.1–0.5 MΩ; Microprobes) was inserted into the right mandibular canal to access the IAN, with a reference electrode placed on the peripheral skin close to the stimulation site. IAN was electrically stimulated (pulse width, 100 μs; frequency, 1 Hz; intensity, 1 mA) using a stimulus isolator (FE-180; AD instruments).

##### Data processing and analysis

EMG data was analyzed using Labchart 8 software (AD Instruments). The reflex latency was determined by calculating the time from the onset of the optogenetic or electrical stimulus to the onset of the reflex. The reflex duration was measured from the onset to the end of the reflex, while amplitude was defined as the peak-to-peak amplitude of the elicited EMG trace.

##### Behavioral analysis of intradental neuron activation using chemogenetics

*Scn10a*-Cre; CAG-LSL-Gq-DREADD KI mice and WT controls were habituated individually in a Plexiglas acrylic chamber (3.78^''^ inner diameter x 6^''^ height) equipped with a custom built controllable rotating stage. A camera was positioned horizontally facing the stage to capture mouse behaviors and facial changes (Logitech 920x, 1080P, 30 fps). Mice were placed in the testing chamber directly from their home cage and video recorded to capture natural behaviors. The stage was gently rotated as needed to ensure we were obtaining video footage with the face directed forward the camera. Frames where the animal was facing away or during rotation were removed from videos. Habituation took place for at least three sessions. Following habituation, each animal underwent a total of 4 experimental paradigms on separate days. The same cohort of mice was assayed for all conditions for each genotype. For baseline recordings, animals were placed directly from their home cage and recorded. This enabled us to categorize the major baseline animal behaviors in our chamber paradigm: ***locomotion*** (movement involving turning or repositioning within the chamber while on all four paws, ***rearing*** (transitioning from sitting/standing position with all paws on the ground to standing on hindpaws, or vice versa), ***grooming*** (body grooming consisting of licking the body below the neck, or face grooming consisting of using the paws to groom the face, head, mouth, and/or ears), and ***resting*** (mouse either remains in place with minor movements or is completely motionless, with ears upright and facing forward). To evaluate behavioral responses to global peripheral *Scn10a*+ neuron activation, chemogenetic activation of Gq-DREADD was achieved using clozapine N-oxide (CNO) (Bioteche, #4936) suspended in 1X PBS. CNO was injected intraperitoneally at a dose of 0.1 mg/kg, using 31 gauge BD insulin syringes on gently restrained mice. Following injection, mice were returned to their home cages for a 25 min acclimation period before being placed in the recording chamber and filmed enabling capture and analysis of 10 min of recording. *Scn10a*-cre; CAG-LSL-Gq-DREADD KI mice exhibited pain-related behaviors which were used to categorize ***pain*** (postural hunching, orofacial grimace consisting of ears pulled apart and back from baseline position and/or orbital tightening). We then applied LabGym^34^, a recently developed automated tool for identifying and quantifying user-defined behaviors, for subsequent analysis. First, we selected 50 frames that were generated from video recordings using LabGym and annotated the mice in these frames with Roboflow (Roboflow: Computer vision tools for developers and enterprises). We used the annotated frames to train a LabGym Detector to detect the mice in all video recordings. Next, we used the trained Detector to generate behavior examples from video recordings and sort them into different behavior categories. The sorted behavior examples were then used to train a LabGym Categorizer for behavior classification. Finally the trained Detector and Categorizer were used to automatically identify and quantify the identified behaviors (locomotion, rearing, grooming, resting, and pain). Experimenters were blinded to conditions and genotypes within each of the analyzed recordings. To assess behavior in response to selective chemogenetic activation of intradental neurons, mice were briefly anesthetized via isoflurane administration (4% for induction and 1.5–2% for maintenance using a SomnoSuite Low-Flow Anesthesia System (Kent Scientific) administered through a secured nose cone. Body temperature was maintained using a hand warmer. Access to the mandibular molars was gently achieved via a custom device designed to separate the maxillary and mandibular incisors to open the mouth vertically, followed by oral insertion of surgical retractors horizontally to expose the molars. Shallow occlusal cavitations to unilateral mandibular first and second molars to expose superficial dentin were prepared with a ¼ round carbide dental bur attached to a micromotor drill (variable RPM) followed by application of temporary fillings (Flow-It ALC B2; Pentron) then light cured to seal the preparation for an 8 hr recovery period. Following recovery, mice were again briefly anesthetized using the described procedure to access the mandibular molars, fillings were removed, CNO (0.01 mg/kg solution, 1.5 μL) was directly applied to exposed dentin until fully absorbed, fillings were replaced and light cured, and animals were allowed to wake and acclimate in their home cage for 25 min prior to behavior recording. To rule out potential off target systemic behavioral effects of CNO tooth application, behavior was also examined in response to i.p. injection of an equivalent dose of CNO (0.01 mg/kg) in mice prior to performing the tooth CNO experiment. Data analysis and statistics were performed using LabGym.

##### Optogenetic stimulation of jaw opening reflex

Mice (*Scn10a*-Cre; Ai32, 8–12 weeks) were anesthetized via isoflurane administration (4% for induction and 0.5–1.5% for maintenance) using a SomnoSuite Low-Flow Anesthesia System (Kent Scientific) administered through a secured nose cone. Mice were held at a level of anesthesia to achieve stable breathing marked by no jaw movement at baseline. Recordings were performed via TTL trigger (Doric, OTPG8) for the synchronization of laser stimulation with frame acquisition using a camera (FLIR, Blackfly S camera) aimed at the opening of the oral cavity. An optical fiber (500 μm, NA 0.63, 5 mm length, Goldstone Scientific) was directed at 90° to target oral cavity tissues to deliver blue (470 ± 6 nm, average intensity 564.8 mW/cm^2^) or green light (545 ± 6 nm, average intensity 548.8 mW/cm^2^) generated by LED light sources (UHT-P-470-SR or UHT-P-545-SR, Goldstone Scientific). Pulses were delivered in alternating trains of green and blue light (10 pulses, 0.5 Hz, 1 s). Intradental HTMRs were selectively targeted by directing the optical fiber at the maxillary molar 1 (m1). To ensure the jaw opening was specific to activation of *Scn10a*+ intradental HTMRs, the stimulation paradigm was repeated for two adjacent tissues, the hard palate and buccal vestibule. The camera was configured to record in secondary trigger mode through a Python script from the following repository: https://github.com/neurojak/pySpinCapture. The field of view was illuminated using an LED array (850 nm, CMIR-110, CM Vision) and the lens was equipped with an IR filter (Edmund Optics, Stock #21–697) to avoid capturing light from the optical fiber. Video was acquired at 170 Hz, and the trigger source for the alignment of video with light stimulation was sampled at 10 kHz. A DeepLabCut (CITE) network was trained to detect the position of the top and bottom incisors of the mouse to measure the amplitude of the mandibular deflections. The Euclidean distance between incisors was calculated and aligned to the laser light trace with sub-millisecond precision in Python.

##### Visualization of *Scn10a*+ neuron central projections

The animal was perfused with 1X PBS (phosphate-buffered saline) followed by 4% paraformaldehyde (PFA) to fix the neural tissue, ensuring preservation of the brain’s structural integrity. After perfusion, the brain was carefully dissected out while keeping the brainstem intact, a crucial step for any subsequent analyses involving brainstem structures. Once dissected, the brain was immersed in 4% PFA for post-fixation overnight, allowing complete tissue fixation. This step is vital for maintaining cellular details and preventing degradation. After fixation, the brain was transferred to a solution of 30% sucrose in 1X PBS, which functions as a cryoprotectant. The brain remains in this solution until it sank, indicating sufficient infiltration (usually about 2–3 days). For long-term preservation, the brain was embedded in an OCT (optimal cutting temperature) compound within a mold, labeled with the sample name, date, and experiment details, and then stored at −80°C until sectioning and further analysis. For sectioning, we set a thickness of 50 μm for each section and collected the serial sections onto gelatin-coated slides. The slides were stored at −80°C until they were ready for imaging and visualization of YFP using confocal microscopy (Olympus FV3000, Evident Scientific, Inc.).

### QUANTIFICATION AND STATISTICAL ANALYSIS

Statistical analyses were performed using GraphPad prism. Statistical methods, error bars, and sample sizes were described in the figure legends for individual experiments. Broadly, ANOVA with post hoc analysis was performed for multi-group comparisons, whereas t test (paired or unpaired as indicated) was used to compare two groups. Statistical significance was defined as *p* values < 0.5.

## Supplementary Material

1

2

3

4

5

6

Supplemental information can be found online at https://doi.org/10.1016/j.celrep.2025.116017.

## Figures and Tables

**Table:** KEY RESOURCES TABLE

REAGENT or RESOURCE	SOURCE	IDENTIFIER
Antibodies		
Rabbit anti-RFP (1:500)	Rockland	600-401-379; RRID:AB_2209751
Chicken anti-GFP (1:1000)	Life Technologies	PA1-9533; RRID:AB_1074893
Sheep anti-GFP/eYFP (1:500)	Bio-Rad	4747-1051; RRID:AB_619712
Rat anti-CD45 (1:500)	BioLegend	103101; RRID:AB_312966
Goat anti-rabbit (Alexa Fluor 568)	Life Technologies	A11011; RRID:AB_143157
Goat anti-chicken (Alexa Fluor 647)	Life Technologies	A21449; RRID:AB_2535866
Donkey anti-sheep (Alexa Fluor 488)	Life Technologies	A11015; RRID:AB_141362
Donkey anti-rat (Alexa Fluor 647)	Invitrogen	A48272; RRID:AB_2893138
Bacterial and virus strains		
ssAAV-9/2-hEF1a-iCre-WPRE-bGHp(A) (Physical titer: 8.1 × 10E12 vg/mL)	University of Zurich Viral Vector Facility VVF, Zurich, CH	v225-9
ssAAV-9/2-hSyn1-chI-mCherry_2A_iCre-WPRE-SV40p(A) (Physical titer: 5.6 × 10E12 vg/mL)	University of Zurich Viral Vector Facility VVF, Zurich, CH	v147-9
AAV9-CAG-FLEX-tdT (Physical titer: 1-9 × 10E13 vg/mL)	Vigene	Discontinued
pAAV-EF1α-Brainbow-invert tagBFP-eYFP-wPRE (Physical titer: 2.2 × 10E13 vg/mL)	Addgene	#V120025
pAAV-Ef1a-Brainbow/mCherry/mTFP-WPRE (Physical titer: 2.2 × 10E13 vg/mL)	Addgene	#V160749
ssAAV-6(F129L)/2-hEF1a-iCre-WPRE-bGHp(A) (Physical titer: 7.2 × 10E12 vg/mL)	University of Zurich Viral Vector Facility VVF, Zurich, CH	#225-6(F129L)/2
Chemicals, peptides, and recombinant proteins		
Cholera Toxin Subunit B (Recombinant), Alexa Fluor^™^ 647 Conjugate	ThermoFisher	C34778
Isofluorane	MWI Animal Health	501017
Normal Donkey Serum	Millipore Sigma	S30-M
Normal Goat Serum	Millipore Sigma	S26-M
Paraformaldehyde (PFA) fixative	Electron Microscopy Sciences	15714-S
5% silk fibroin	Advanced BioMatrix	5154-20ML
Flow-It^™^ ALC^™^	Pentron	Shade B2
Deposited data		
Custom code for analysis	Zenodo	https://doi.org/10.5281/zenodo.15643732
Critical commercial assays		
Hybridization Chain Reaction (HCR) version 3 kit	Molecular Instruments	N/A
RNAscopeTM Multiplex Fluorescent Detection Reagents version 2	Advanced Cell Diagnostics, Inc	323110
Experimental models: Organisms/strains		
Mouse: Ai95(RCL-GCaMP6f)-D (C57BL/6J)	JAX (The Jackson Laboratory)	Stock no. 028865; RRID:IMSR_JAX:028865
Mouse: Piezo2^lox/lox^; Tac1-tagRFP-2a-TVA line	Szczot et al.^19^	N/A
Mouse: Snap25-LSL-2A-EGFP-D	JAX	Stock no. 021879; RRID:IMSR_JAX:021879
Mouse: Na_v_1.8-Cre	JAX	Stock no. 036564; RRID:IMSR_JAX:036564
Mouse: CAG-LSL-Gq-DREADD	JAX	Stock no. 026220; RRID:IMSR_JAX:026220
Mouse: C57BL/6J	JAX	Stock no. 000664; RRID:IMSR_JAX:000664
Mouse: Ai65(RCF-tdT)	JAX	Stock no. 032864; RRID:IMSR_JAX:032864
Mouse: Ai32(RCL-ChR2(H134R)/EYFP)	JAX	Stock no. 024109; RRID:IMSR_JAX:024109
Mouse: S100b-Cre	N/A	Gift from Dr. Nicholas Ryba
Oligonucleotides		
Mm-S100b-C1	Advanced Cell Diagnostics, Inc	431731
Mm-Bmpr1b-C2	Advanced Cell Diagnostics, Inc	533941-C2
Mm-Smr2-C2	Advanced Cell Diagnostics, Inc	538931-C2
*S100b*	Molecular Instruments	PRA454
*Scn10a*	Molecular Instruments	GenBank: NM_009134.3
*Calca*	Molecular Instruments	PRB722
*Mrgprd*	Molecular Instruments	PRO964
*GCaMP6f*	Molecular Instruments	RTE507
*Fxyd2*	Molecular Instruments	PRB740
*TdTomato*	Molecular Instruments	PRF678
*Piezo2*	Molecular Instruments	GenBank: NM_001039485.4
*Tubb3*	Molecular Instruments	PRO586
*eYFP*	Molecular Instruments	PRE356
Software and algorithms		
MATLAB	Mathworks	https://www.mathworks.com/products.html
Fiji	NIH	https://doi.org/10.1038/nmeth.2019
LabGym	N/A	https://github.com/umyelab/LabGym
Python	The Python Software Foundation	https://www.python.org/
LabScribe V4 software	IWORX	https://iworx.com/labscribe-software-download/?v=0b3b97fa6688
GraphPad Prism software	GraphPad Software, Inc	Graphpad.com
Labchart 8 software	AD Instruments	https://www.adinstruments.com/support/software?srsltid=AfmBOorppio3YpxwdPVuEd4VtTPwx5OpYccXeLsYT1kR_-D5PEvArJyw

## Highlights

- *In vivo* calcium imaging enables real-time monitoring of intradental neurons
- Intradental neurons are mechano-nociceptors that respond to superficial tooth damage
- Activation of intradental neurons elicits pain identified via LabGym analysis
- Activation of intradental neurons triggers a protective jaw-opening reflex

## References

[1] Xu K-H, Li L, Jia S-L, Li Q, Hao J-X, Ma S, He Z-K, Wan Q-Q, Cai Y-F, Li Z-T, (2023). Association of Tooth Loss and Diet Quality with Acceleration of Aging: Evidence from NHANES. Am. J. Med 136, 773–779.e4. 10.1016/j.amjmed.2023.04.008. 37075877

[2] Koka S, and Gupta A (2018). Association between missing tooth count and mortality: A systematic review. J. Prosthodont. Res 62, 134–151. 10.1016/j.jpor.2017.08.003. 28869174

[3] Ghitani N, Barik A, Szczot M, Thompson JH, Li C, Le Pichon CE, Krashes MJ, and Chesler AT (2017). Specialized Mechanosensory Nociceptors Mediating Rapid Responses to Hair Pull. Neuron 95, 944–954. e4. 10.1016/j.neuron.2017.07.024. 28817806 PMC5599122

[4] Barik A, Thompson JH, Seltzer M, Ghitani N, and Chesler AT (2018). A Brainstem-Spinal Circuit Controlling Nocifensive Behavior. Neuron 100, 1491–1503.e3. 10.1016/j.neuron.2018.10.037. 30449655

[5] Henry MA, and Hargreaves KM (2007). Peripheral Mechanisms of Odontogenic Pain. Dent. Clin. North Am 51, 19–44. 10.1016/j.cden.2006.09.007. 17185058

[6] Renton T (2011). Dental (Odontogenic) Pain. Rev. Pain 5, 2–7. 10.1177/204946371100500102. PMC4590084 26527224

[7] Slade GD (2001). Epidemiology of dental pain and dental caries among children and adolescents. Community Dent. Health 18, 219–227. 11789699

[8] Byers MR, and Cornel LM (2018). Multiple complex somatosensory systems in mature rat molars defined by immunohistochemistry. Arch. Oral Biol 85, 84–97. 10.1016/j.archoralbio.2017.09.007. 29035722

[9] Nguyen MQ, Wu Y, Bonilla LS, von Buchholtz LJ, and Ryba NJP (2017). Diversity amongst trigeminal neurons revealed by high throughput single cell sequencing. PLoS One 12, e0185543. 10.1371/journal.pone.0185543. 28957441 PMC5619795

[10] von Buchholtz LJ, Ghitani N, Lam RM, Licholai JA, Chesler AT, and Ryba NJP (2021). Decoding Cellular Mechanisms for Mechanosensory Discrimination. Neuron 109, 285–298.e5. 10.1016/j.neuron.2020.10.028. 33186546 PMC9909446

[11] Närhi MV, Hirvonen TJ, and Hakumäki MO (1982). Activation of intradental nerves in the dog to some stimuli applied to the dentine. Arch. Oral Biol 27, 1053–1058. 10.1016/0003-9969(82)90011-5. 6963884

[12] von Buchholtz LJ, Lam RM, Emrick JJ, Chesler AT, and Ryba NJP (2020). Assigning transcriptomic class in the trigeminal ganglion using multiplex in situ hybridization and machine learning. Pain 161, 2212–2224. 10.1097/j.pain.0000000000001911. 32379225 PMC7606614

[13] Sharma N, Flaherty K, Lezgiyeva K, Wagner DE, Klein AM, and Ginty DD (2020). The emergence of transcriptional identity in somatosensory neurons. Nature 577, 392–398. 10.1038/s41586-019-1900-1. 31915380 PMC7307422

[14] Usoskin D, Furlan A, Islam S, Abdo H, Lönnerberg P, Lou D, Hjerling-Leffler J, Haeggström J, Kharchenko O, Kharchenko PV, (2015). Unbiased classification of sensory neuron types by large-scale single-cell RNA sequencing. Nat. Neurosci. 18, 145–153. 10.1038/nn.3881. 25420068

[15] Servin-Vences MR, Lam RM, Koolen A, Wang Y, Saade DN, Loud M, Kacmaz H, Frausto S, Zhang Y, Beyder A, (2023). PIEZO2 in somatosensory neurons controls gastrointestinal transit. Cell 186, 3386–3399.e15. 10.1016/j.cell.2023.07.006. 37541196 PMC10501318

[16] Lam RM, von Buchholtz LJ, Falgairolle M, Osborne J, Frangos E, Servin-Vences MR, Nagel M, Nguyen MQ, Jayabalan M, Saade D, (2023). PIEZO2 and perineal mechanosensation are essential for sexual function. Science 381, 906–910. 10.1126/science.adg0144. 37616369 PMC11418610

[17] Qi L, Iskols M, Shi D, Reddy P, Walker C, Lezgiyeva K, Voisin T, Pawlak M, Kuchroo VK, Chiu I, (2023). A DRG genetic toolkit reveals molecular, morphological, and functional diversity of somatosensory neuron subtypes. preprint at bioRxiv 10.1101/2023.04.22.537932. PMC10947841 38442711

[18] Yarmolinsky DA, Peng Y, Pogorzala LA, Rutlin M, Hoon MA, and Zuker CS (2016). Coding and Plasticity in the Mammalian Thermosensory System. Neuron 92, 1079–1092. 10.1016/j.neuron.2016.10.021. 27840000 PMC5145739

[19] Szczot M, Liljencrantz J, Ghitani N, Barik A, Lam R, Thompson JH, Bharucha-Goebel D, Saade D, Necaise A, Donkervoort S, (2018). PIEZO2 mediates injury-induced tactile pain in mice and humans. Sci. Transl. Med 10, eaat9892. 10.1126/scitranslmed.aat9892. 30305456 PMC6875774

[20] Virtanen A, Närhi M, Huopaniemi T, and Hirvonen T (1983). Thresholds of intradental A-and C-nerve fibres in the cat to electric current pulses of different duration. Acta Physiol. Scand 119, 393–398. 10.1111/j.1748-1716.1983.tb07355.x. 6666620

[21] Chen E, and Abbott PV (2009). Dental Pulp Testing: A Review. Int. J. Dent 2009, 365785. 10.1155/2009/365785. 20339575 PMC2837315

[22] Emrick JJ, von Buchholtz LJ, and Ryba NJP (2020). Transcriptomic Classification of Neurons Innervating Teeth. J. Dent. Res 99, 1478–1485. 10.1177/0022034520941837. 32702253 PMC7684839

[23] Qi L, Iskols M, Shi D, Reddy P, Walker C, Lezgiyeva K, Voisin T, Pawlak M, Kuchroo VK, Chiu IM, (2024). A mouse DRG genetic toolkit reveals morphological and physiological diversity of somatosensory neuron subtypes. Cell 187, 1508–1526.e16. 10.1016/j.cell.2024.02.006. 38442711 PMC10947841

[24] Bhuiyan SA, Xu M, Yang L, Semizoglou E, Bhatia P, Pantaleo KI, Tochitsky I, Jain A, Erdogan B, Blair S, (2024). Harmonized cross-species cell atlases of trigeminal and dorsal root ganglia. Sci. Adv 10, eadj9173. 10.1126/sciadv.adj9173. 38905344 PMC11804847

[25] Wanachantararak S, Ajcharanukul O, Vongsavan N, and Matthews B (2016). Effect of cavity depth on dentine sensitivity in man. Arch. Oral Biol 66, 120–128. 10.1016/j.archoralbio.2016.02.015. 26945170

[26] Byers MR, and Calkins DF (2021). Trigeminal sensory nerve patterns in dentine and their responses to attrition in rat molars. Arch. Oral Biol 129, 105197. 10.1016/j.archoralbio.2021.105197. 34146928 PMC8364492

[27] Murthy SE, Loud MC, Daou I, Marshall KL, Schwaller F, Kühne-mund J, Francisco AG, Keenan WT, Dubin AE, Lewin GR, and Patapoutian A (2018). The mechanosensitive ion channel Piezo2 mediates sensitivity to mechanical pain in mice. Sci. Transl. Med 10, eaat9897. 10.1126/scitranslmed.aat9897. 30305457 PMC6709986

[28] Akopian AN, Souslova V, England S, Okuse K, Ogata N, Ure J, Smith A, Kerr BJ, McMahon SB, Boyce S, (1999). The tetrodotoxin-resistant sodium channel SNS has a specialized function in pain pathways. Nat. Neurosci 2, 541–548. 10.1038/9195. 10448219

[29] Stirling LC, Forlani G, Baker MD, Wood JN, Matthews EA, Dickenson AH, and Nassar MA (2005). Nociceptor-specific gene deletion using heterozygous NaV1.8-Cre recombinase mice. Pain 113, 27–36. 10.1016/j.pain.2004.08.015. 15621361

[30] Nassar MA, Stirling LC, Forlani G, Baker MD, Matthews EA, Dickenson AH, and Wood JN (2004). Nociceptor-specific gene deletion reveals a major role for Nav1.7 (PN1) in acute and inflammatory pain. Proc. Natl. Acad. Sci. USA 101, 12706–12711. 10.1073/pnas.0404915101. 15314237 PMC515119

[31] Närhi M (1990). The neurophysiology of the teeth. Dent. Clin. North Am 34, 439–448. 2197120

[32] Guo C, Jiang H, Huang C-C, Li F, Olson W, Yang W, Fleming M, Yu G, Hoekel G, Luo W, and Liu Q (2023). Pain and itch coding mechanisms of polymodal sensory neurons. Cell Rep 42, 113316. 10.1016/j.celrep.2023.113316. 37889748 PMC10729537

[33] Rossi HL, See LP, Foster W, Pitake S, Gibbs J, Schmidt B, Mitchell CH, and Abdus-Saboor I (2020). Evoked and spontaneous pain assessment during tooth pulp injury. Sci. Rep 10, 2759. 10.1038/s41598-020-59742-5. 32066827 PMC7026088

[34] Hu Y, Ferrario CR, Maitland AD, Ionides RB, Ghimire A, Watson B, Iwasaki K, White H, Xi Y, Zhou J, and Ye B (2023). LabGym: Quantification of user-defined animal behaviors using learning-based holistic assessment. Cell Rep. Methods 3, 100415. 10.1016/j.crmeth.2023.100415. 37056376 PMC10088092

[35] Goss K, Bueno-Junior LS, Stangis K, Ardoin T, Carmon H, Zhou J, Satapathy R, Baker I, Jones-Tinsley CE, Lim MM, (2024). Quantifying social roles in multi-animal videos using subject-aware deep-learning. Preprint at bioRxiv. 10.1101/2024.07.07.602350.

[36] Närhi M, Virtanen A, Hirvonen T, and Huopaniemi T (1983). Comparison of electrical thresholds of intradental nerves and jaw-opening reflex in the cat. Acta Physiol. Scand 119, 399–403. 10.1111/j.1748-1716.1983.tb07356.x. 6666621

[37] Perry SK, and Emrick JJ (2024). Trigeminal somatosensation in the temporomandibular joint and associated disorders. Front. Pain Res 5, 1374929. 10.3389/fpain.2024.1374929. PMC11111860 38784786

[38] Brannstrom M (1986). The hydrodynamic theory of dentinal pain: sensation in preparations, caries, and the dentinal crack syndrome. J. Endod 12, 453–457. 10.1016/S0099-2399(86)80198-4. 3465849

[39] Dong WK, Chudler EH, and Martin RF (1985). Physiological properties of intradental mechanoreceptors. Brain Res 334, 389–395. 10.1016/0006-8993(85)90239-2. 3873270

[40] Jones J, Correll DJ, Lechner SM, Jazic I, Miao X, Shaw D, Simard C, Osteen JD, Hare B, Beaton A, (2023). Selective Inhibition of NaV1.8 with VX-548 for Acute Pain. N. Engl. J. Med 389, 393–405. 10.1056/NEJMoa2209870. 37530822

[41] Lee PR, Kim J, Rossi HL, Chung S, Han SY, Kim J, and Oh SB (2023). Transcriptional profiling of dental sensory and proprioceptive trigeminal neurons using single-cell RNA sequencing. Int. J. Oral Sci 15, 45. 10.1038/s41368-023-00246-z. 37749100 PMC10519964

[42] Bleicher F (2014). Odontoblast physiology. Exp. Cell Res 325, 65–71. 10.1016/j.yexcr.2013.12.012. 24361392

[43] Abraira VE, and Ginty DD (2013). The sensory neurons of touch. Neuron 79, 618–639. 10.1016/j.neuron.2013.07.051. 23972592 PMC3811145

[44] Lynch CD, and McConnell RJ (2002). The cracked tooth syndrome. J. Can. Dent. Assoc 68, 470–475. 12323102

[45] Funaki Y, Hiranuma M, Shibata M, Kokai S, and Ono T (2014). Effects of nasal obstruction on maturation of the jaw-opening reflex in growing rats. Arch Oral Biol 59, 530–538. 10.1016/j.archoralbio.2014.02.013. 24658014

[46] Uchima Koecklin KH, Hiranuma M, Kato C, Funaki Y, Kataguchi T, Yabushita T, Kokai S, and Ono T (2016). Unilateral Nasal Obstruction during Later Growth Periods Affects Craniofacial Muscles in Rats. Front Physiol 7, 669. 10.3389/fphys.2016.00669. 28119621 PMC5222814

