# Supplementary material for: Intradental mechano-nociceptors serve as sentinels that prevent tooth damage
Source: Cell Rep. Author manuscript; Available in PMC 2025 Sep 17. (PMC12442953; doi:10.1016/j.celrep.2025.116017)
Supplement: 1 [file NIHMS2107249-supplement-1.pdf]

Cell Reports, Volume *44*

Supplemental information

Intradental mechano-nociceptors serve as sentinels that prevent tooth damage

Elizabeth A. Ronan, Akash R. Gandhi, Karin H. Uchima Koecklin, Yujia Hu, Shuhao Wan, Brian S.C. Constantinescu, Mak E. Guenther, Maximilian Nagel, Ling-Yu Liu, Aditi Jha, Leen Dakhilalla, Kaitlyn J. Blumberg, Isaac T. Berthaume, Tomer Stern, Kevin P. Pipe, Bing Ye, Peng Li, and Joshua J. Emrick

SUPPLEMENTAL FIGURES AND LEGENDS

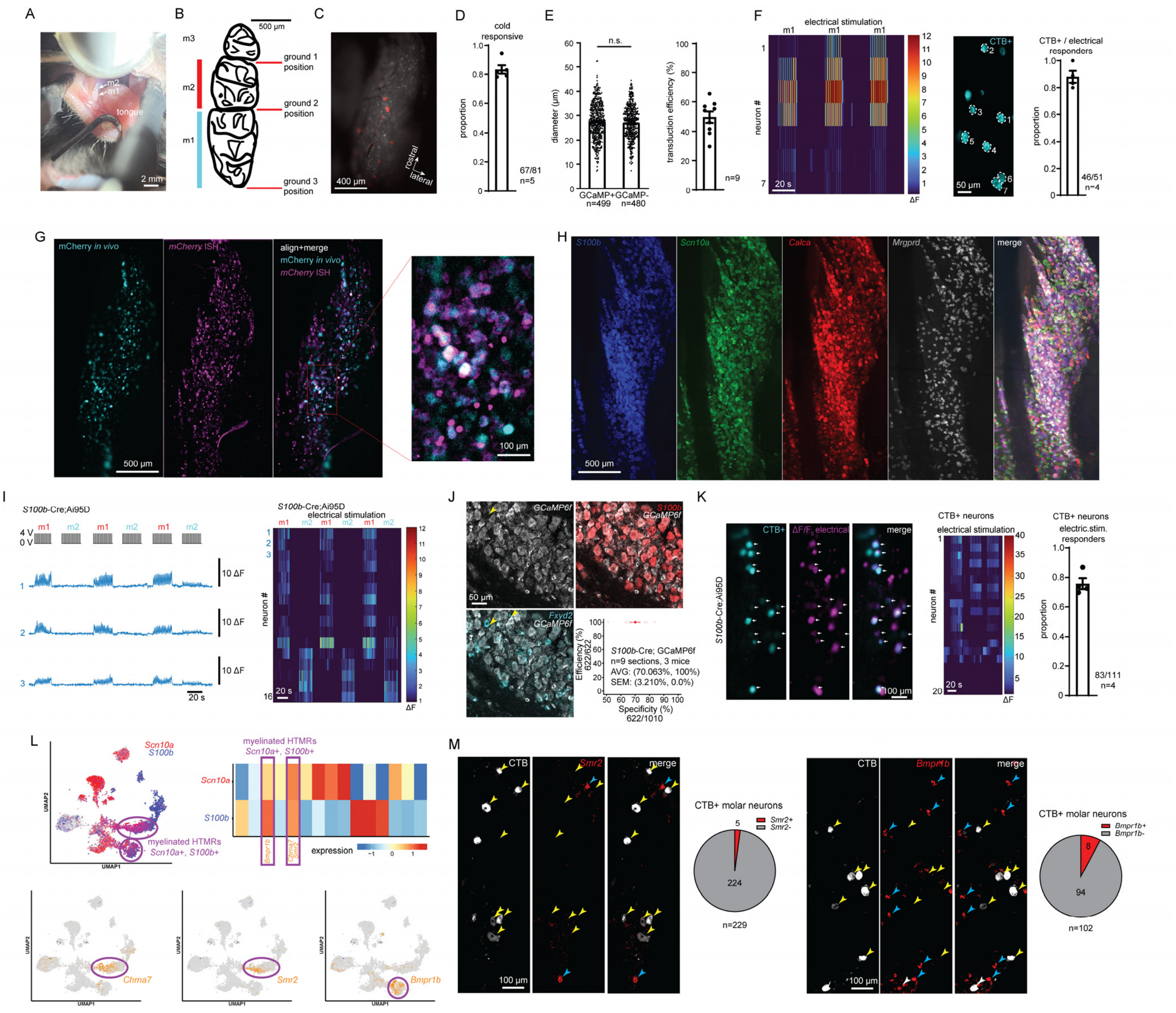

Figure S1. Additional data on functional imaging and molecular characterization of intradental neurons, and validation and functional imaging using *S100b*-Cre driver line. Related to Figure 1.

(A) Snapshot image showing the oral cavity field of view as viewed by operator in a head fixed mouse during in vivo imaging. Molar 1 (m1), molar 2 (m2), and the protruded tongue are annotated. Scale bar, 2 mm.

(B) Illustrative schematic showing locations of a fine wire cathode (-) placement during successive rounds of molar voltage stimulation. To identify intradental trigeminal neurons innervating a single molar, a fine wire anode (+) was held on the occlusal surface of the molar, while the cathode was positioned in the lingual gingiva. Delivery of voltage pulses to a single molar was repeated for a total of 3 times while varying the location of the cathode to each of the three ground positions shown. Molar 1 (m1), molar 2 (m2) and molar 3 (m3) are annotated. Scale bar, 500 μm.

(C) Epifluorescent image of a trigeminal ganglion in a AAV9-Cre injected Ai95D mouse demonstrating the localization of neurons responding to voltage pulses (red). In vivo visualization of the trigeminal ganglion surface is achieved via a cranial window preparation and a modified stereotax to fix the head (see Methods). Trigeminal neurons responding to molar stimulation are located in the caudal, mandibular division of the ganglion. Scale bar, 400 μm.

(D) Data on neonatal AAV transduction of trigeminal sensory neurons. Left: Bar graph showing the mean diameter of GCaMP+ versus GCaMP- trigeminal neurons from adult Ai95(RCL-GCaMP6f)-D (Ai95D) mice injected postnatally (P0-P3) with AAV9-Cre. The expression of GCaMP was amplified with ISH and quantified in nonsequential sections from 6 TG taken from 3 mice. Plotted individual data points represent single somal diameters. Bar shows the mean value, and error bars indicate the SEM. No significant differences were observed with $p > 0.05$ using one-way ANOVA with Tukey’s correction. Right: Bar graph showing the transduction efficiency of GCaMP from adult Ai95(RCL-GCaMP6f)-D (Ai95D) mice injected postnatally (P0-P3) with AAV9-Cre. The expression of GCaMP was examined by ISH. Plotted individual data points represent single somal diameters. Bar shows the mean value, and error bars indicate the SEM. Quantifications are from 9 TG sections taken from 3 mice.

(E) Bar graph depicting the proportion of intradental neurons that respond to cold stimulation. Plotted individual data points represent the proportion of cold responsive/intradental neurons identified in each experiment. Bar shows the mean, and error bars indicate the SEM. n = 5 mice, comprising 81 total cells. $p > 0.05$ using unpaired t test.

(F) Retrograde labeling marks intradental neurons that are activated by electrical stimulation of the molars. Retrograde tracer CTB-AF647 was applied to small cavitations in a single mandibular molar (m1) 16 hr prior to functional imaging in a AAV9-Cre injected Ai95D mouse. Example heatmap showing calcium responses of CTB+ neurons to voltage pulses. Scale bar, 20 s. Epifluorescent image of corresponding CTB-647 retrograde-labeled intradental neurons shown in G. Scale bar, 50 µm. Bar graph showing the proportion of TG neurons responding to ≥2 voltage pulses that are CTB+. Bar shows the mean and error bars indicate the SEM. n = 4 mice. CTB, cholera toxin B-subunit.

(G-H) Example images of alignment of functional images with ISH images. (G) Dorsal view of the mCherry-positive neurons in the TG in vivo (cyan) and excised ganglion after whole-mount ISH against *mCherry* (magenta). Images were aligned using a combination of Demon's algorithm and Bigwarp (see STAR Methods) for image registration via MatLab (right panel). Scale bar, 500µm. Inset of the field demonstrates overlap (white). TGs were from Ai95(RCL-GCaMP6f)-D (Ai95D) mice injected postnatally (P0-P3) with AAV9-hSyn1-mCherry-2A-iCre, enabling mCherry expression to serve as guideposts to align functional imaging with ISH images. Scale bar, 100 µm. (H) Example images showing multiplexed whole-mount ISH staining following alignment to in vivo GCaMP6f fluorescence. Probes: s100 calcium-binding protein B (*S100b*, blue), alpha-Nav1.8 (*Scn10a*, green), calcitonin gene-related peptide (CGRP; *Calca*, red), mas-related G protein-coupled receptor member D (*Mrgprd*, white). Scale bar, 500 µm (left).

(I) Example traces and related heatmap showing electrical responses in *S100b*-Cre; Ai95D transgenic mice. Vertical scale bars, 10 ΔF. Horizontal scale bars, 20 s.

(J) Validation of the *S100b*-Cre driver line. Representative ISH images for a single section of the trigeminal ganglion taken from *S100b*-Cre; Ai95D mice. Probes (left to right): *GCaMP6f*, s100 calcium-binding protein B (*S100b*), FXYD domain-containing ion transport regulator 2 (*Fxyd2*). Yellow arrows indicate *Fxyd2*(+)/*GCaMP6f*(-)/*S100b*(-) cells (left and right panels). Scale bar: 50 μm. Line graph depicts summary of the specificity and efficiency of *GCaMP6f* expression in our transgenic *S100b*-Cre; Ai95D mice. Specificity refers to the percentage of *GCaMP6f*+ cells that are S100b+. Efficiency refers to the percentage of *S100b*+ cells that express *GCaMP6f*+. n = 9 sections from 3 mice.

(K) Data obtained from *S100b*-Cre; Ai95D mice. Retrograde labeling marks intradental neurons that are activated by electrical stimulation of the molars. Retrograde tracer CTB-AF555 was applied to small cavitations in mandibular molars (m1/m2) 16 hr prior to functional imaging. Sample images depicting overlap of CTB labeling with Ca^{2+} response (ΔF) of neurons responding to applied voltage pulses. Scale bar, 50μm. Left panel: CTB labeling; Middle panel: single frame (ΔF) from recording during response to voltage pulse; Right panel: merge. Example heatmap showing responses of combined m1/m2 CTB+ neurons to voltage pulses. Bar graph showing the proportion of CTB+ TG neurons that respond to ≥2 voltage pulses. Plotted individual data points represent the calculated proportion of CTB+ cells that respond to ≥2 voltage pulses for each ganglion. Bar shows the mean and error bars indicate the SEM. n = 4 mice. CTB, cholera toxin B-subunit.

(L-M) Gene expression data from integrated datasets and plots generated from painseq.shinyapps.io (Bhuiyan, A. S., Xu M., et al. doi: 10.1126/sciadv.adj9173).

(L) UMAP projection and heatmap representing gene expression patterns of *Scn10a* (red) and *S100b* (blue). Top left panel: Dots represent cells/nuclei. Clusters represent distinct classes of trigeminal sensory neurons from the atlas. Co-expression of *Scn10a* and *S100b* (purple) is found primarily within two clusters that represent myelinated HTMRs (purple ovals). Top right panel: Heatmap representing dataset from UMAP demonstrating Scn10a and S100b normalized expression across distinct classes of trigeminal sensory neurons from the atlas. Orange and red indicate normalized expression >0. Two classes feature co-expression of Scn10a and S100b >0 (purple rectangles) and represent myelinated HTMRs that are also defined by enriched expression of *Bmpr1b*, *Chrna7*, *Smr2*. Bottom panels: UMAP projection representing gene expression patterns of *Chrna7***,** *Smr2***,** or *Bmpr1b*. Co-expression of *Scn10a* and *S100b* is found primarily within two clusters that represent myelinated HTMRs (purple ovals).

(M) Representative image from retrograde-labeling of intradental neurons using CTB-AF647 tracer followed by ISH. Retrograde tracer CTB-AF647 was applied to small cavitations in maxillary and mandibular molars (m1/m2) 16 hr prior to tissue harvest. The expression of the candidate marker gene *Smr2* or *Bmpr1b* was examined by ISH. Yellow arrow heads indicate CTB-AF647+ intradental neurons. Blue arrowheads indicate cells that are only positive for gene of interest. Scale bars, 100 µm. Pie chart represents counts of CTB-labeled intradental neurons that were positive or negative for *Smr2* (n = 3 mice, comprising 229 retrograde-labeled intradental neurons) or *Bmpr1b* (n=3 mice, comprising 102 retrograde-labeled intradental neurons).

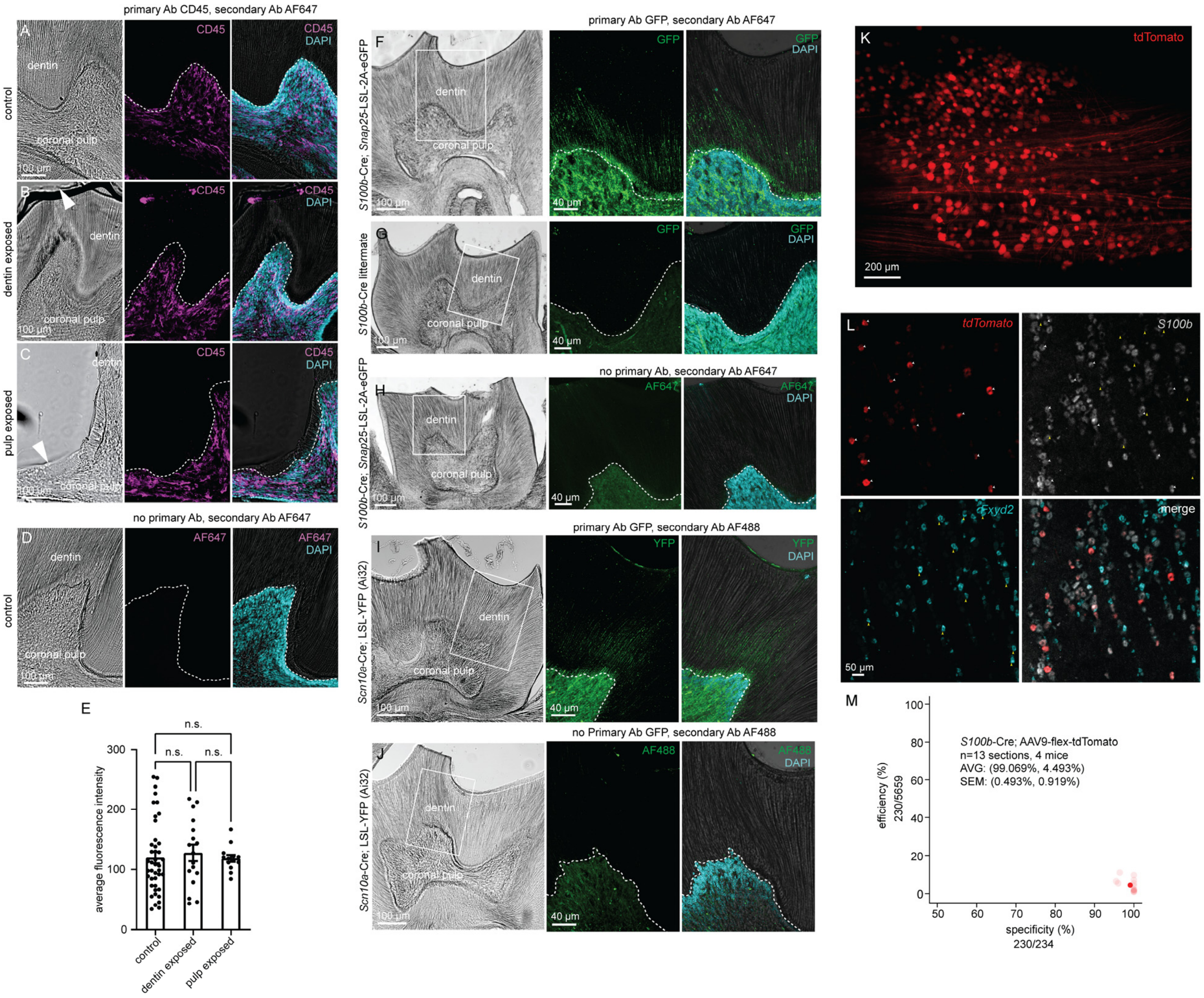

Figure S2. Immunohistochemistry of immune cells in dental pulp; *S100b*-Cre and *Scn10a*-Cre terminal endings in dental pulp and inner dentin; and validation of the S100b-Cre driver line in conjunction with AAV9-flex-tdTdirect trigeminal injections. Related to Figure 2.

(A-C) Representative images of staining for immunoreactivity to CD45 in control, dentin exposed, or pulp exposed molars. Enamel, a fully mineralized structure, has been removed completely by demineralization. (A) Left panel: Brightfield image of control m1 molar cross section, highlighting anatomy of the coronal pulp versus dentin boundary. Middle panel: Immunostaining for CD45. Right panel: Merged image overlaid with DAPI staining labeling

nuclei of pulp cells and brightfield image. Dashed lines indicate the pulp-dentin border. This experiment was repeated in n = 3 mice. Scale bar, 100 μm (B) Left panel: Brightfield image of m1 molar cross section following dentin exposure, highlighting anatomy of the coronal pulp versus dentin boundary. White arrowhead indicates depth of dentin exposure. Middle panel: Immunostaining for CD45. Right panel: Merged image overlaid with DAPI staining labeling nuclei of pulp cells and brightfield image. Dashed lines indicate the pulp-dentin border. This experiment was repeated in n = 3 mice. Scale bar, 100 μm. (C) Left panel: Brightfield image of m1 molar cross section following pulp exposure. White arrowhead indicates exposed pulp. Middle panel: Immunostaining for CD45. Right panel: Merged image overlaid with DAPI staining labeling nuclei of pulp cells and brightfield image. Dashed lines indicate the pulp-dentin border. This experiment was repeated in n = 3 mice. Scale bar, 100 μm

(D) Representative image of immunostaining omitting primary antibody for CD45. Left panel: Brightfield image of m1 molar cross section. Middle panel: Immunostaining shows no AF647+ signal in the absence of the primary antibody. Right panel: Merged image overlaid with DAPI-stained cell nuclei in the pulp and brightfield image. Dashed lines indicates pulp-dentin border. This experiment was repeated in n = 3 mice. Scale bar, 100 μm.

(E) Bar graph depicting the quantified mean fluorescent intensity of CD45 within the pulp across all conditions. Plotted individual data points represent the average intensity for individual tooth pulps. Bar shows the mean, and error bars indicate the SEM. n = 3 mice. $p > 0.05$ using one-way ANOVA with Tukey’s correction.

(F) Representative images of immunostaining for neuronal terminals in demineralized molars from *S100b*-Cre; *Snap25*-LSL-2A-eGFP mice. Enamel, a fully mineralized structure, has been removed completely by demineralization. Left panel: Brightfield image of molar cross section, highlighting anatomy of the coronal pulp versus dentin boundary. Middle panel: Magnified

image of white rectangle. Immunostaining for GFP is localized to *S100b*-positive terminal endings that radiate from the pulp and extend into the dentin. Right panel: Merged image overlaid with DAPI staining labeling nuclei of pulp cells and brightfield image. Dashed lines in indicate the pulp-dentin border. This experiment was repeated in n = 3 mice. Scale bars, 100 µm (left panel) or 40 µm (middle and right panels).

(G) Representative image of *S100b*-Cre; *Snap25*-LSL-2A-eGFP(-) littermate controls showing no GFP+ signal. Enamel, a fully mineralized structure, has been removed completely by demineralization. Left panel: Brightfield image of molar cross section. Middle panel: Magnified image of white rectangle. Right panel: Merged image overlaid with DAPI staining labeling nuclei of pulp cells and brightfield image showing immunostaining for GFP is absent. Dashed lines indicate pulp-dentin border. This experiment was repeated in n = 3 mice. Scale bars, 100 µm (left panel) or 40 µm (middle and right panels).

(H) Representative image of immunostaining omitting primary antibody for *S100b*-Cre; *Snap25*-LSL-2A-eGFP mice. Enamel, a fully mineralized structure, has been removed completely by demineralization. Left panel: Brightfield image of molar cross section. Middle panel: Magnified image of white rectangle. Right panel: Immunostaining shows no GFP+ signal, overlaid with DAPI-stained cell nuclei in the pulp and brightfield image. Dashed lines indicate pulp-dentin border. This experiment was repeated in n = 3 mice. Scale bars, 100 µm (left panel) or 40 µm (middle and right panels)

(I) Representative images of immunostaining for neuronal terminals in demineralized molars from *Scn10a*-Cre; *Ai32* mice. Enamel, a fully mineralized structure, has been removed completely by demineralization. Left panel: Brightfield image of molar cross section, highlighting anatomy of the coronal pulp versus dentin boundary. Middle panel: Magnified image of white rectangle. Immunostaining for YFP is localized to *Scn10a*-positive terminal

endings that radiate from the pulp and extend into the dentin. Right panel: Merged image overlaid with DAPI staining labeling nuclei of pulp cells and brightfield image. Dashed lines indicate the pulp-dentin border. This experiment was repeated in n = 4 mice. Scale bars, 100 µm (left panel) or 40 µm (middle and right panels).

(J) Representative image of immunostaining omitting primary antibody for *Scn10a*-Cre; *Ai32* mice. Enamel, a fully mineralized structure, has been removed completely by demineralization. Left panel: Brightfield image of molar cross section. Middle panel: Magnified image of white rectangle. Right panel: Immunostaining shows no YFP+ signal, overlaid with DAPI-stained cell nuclei in the pulp and brightfield image. Dashed lines indicate pulp-dentin border. This experiment was repeated in n = 4 mice. Scale bars: 100 µm (left panel) or 40 µm (middle and right panels).

(K) Sparse labeling of trigeminal neurons and intradental neuron endings. AAV9-flex-tdTomato was orbitally injected into the trigeminal ganglion to induce tdTomato labeling in neurons of *S100b*-Cre mice. Example whole mount image of the TG validating approach and depicting tdTomato+ positive somas in an *S100b*-Cre mouse following orbital delivery of AAV9-flex-tdT. Scale bar, 200 µm.

(L) Representative ISH images for a single section of the trigeminal ganglion taken from a *S100b*-Cre mouse following orbital injection of AAV9-flex-tdTomato. Probes (left to right): *tdTomato*, s100 calcium-binding protein B (*S100b*), FXYD domain-containing ion transport regulator 2 (*Fxyd2*). Merge: bottom right panel. Green arrow heads indicate particular *Scn10a*+ cells. White arrow heads highlight cells that are co-positive for *S100b* and *tdTomato*. Yellow arrowheads indicate cells that are only positive for *Fxyd2*. Scale bar, 50 µm.

(M) Summary of the specificity and efficiency of TG *tdTomato* expression following AAV9-flex-tdTomato orbital injection in *S100b*-Cre mice. Specificity refers to the percentage of *tdTomato*+ cells that are *S100b*+. Efficiency refers to the percentage of *S100b*+ cells that express *tdTomato*+. n = 13 sections from 4 mice.

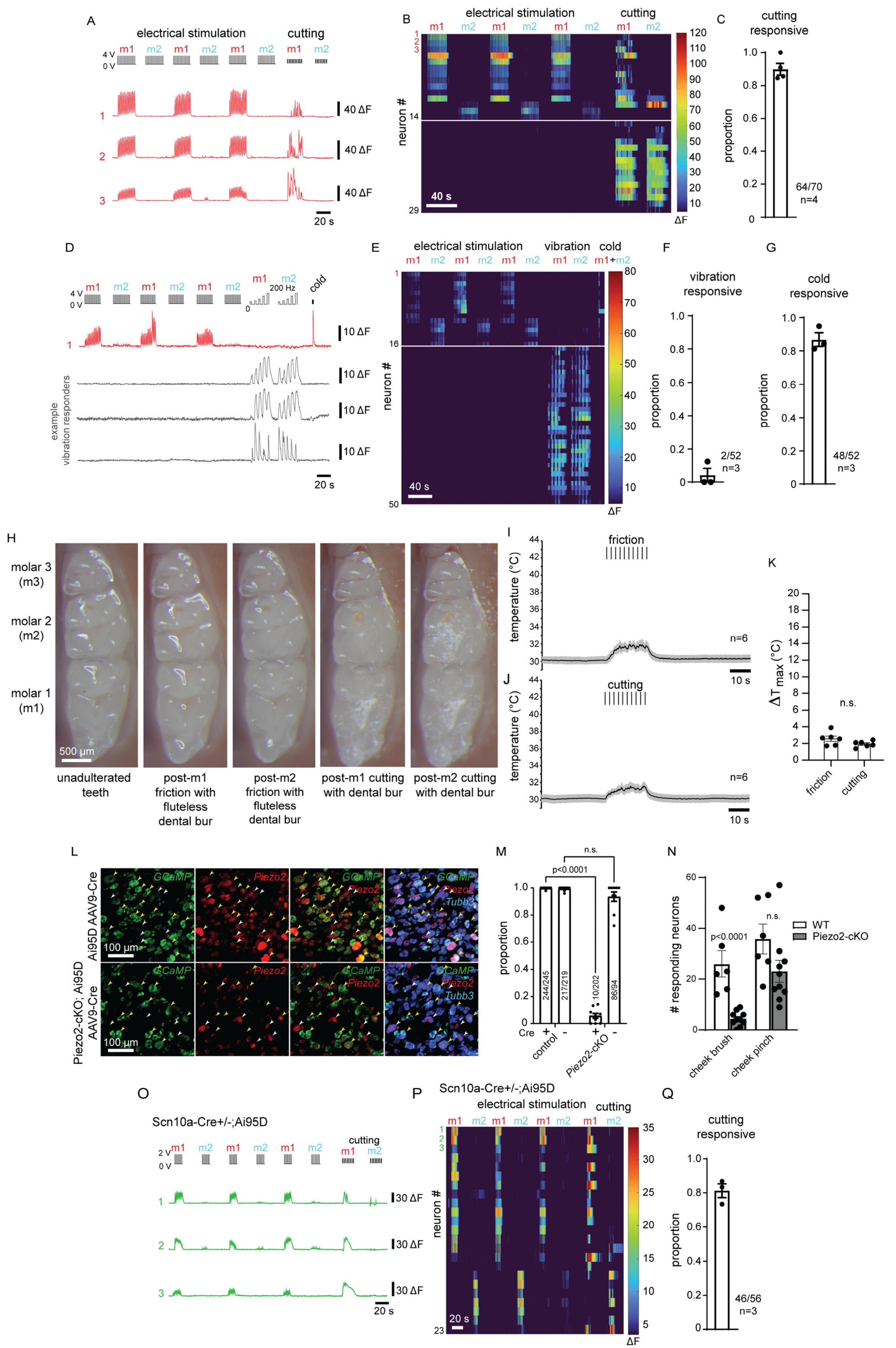
A
electrical stimulation
cutting
m1
m2
40 ΔF
20 s
B
neuron #
40 s
ΔF
C
cutting responsive
proportion
64/70
n=4
D
200 Hz
cold
10 ΔF
example vibration responders
E
vibration
m1+m2
F
vibration responsive
2/52
n=3
G
cold responsive
48/52
H
molar 3 (m3)
molar 2 (m2)
molar 1 (m1)
500 µm
unadulterated teeth
post-m1 friction with fluteless dental bur
post-m2 friction with fluteless dental bur
post-m1 cutting with dental bur
post-m2 cutting with dental bur
I
friction
temperature (°C)
n=6
10 s
J
K
ΔT max (°C)
n.s.
L
Ai95D AAV9-Cre
Piezo2-cKO; Ai95D AAV9-Cre
GCaMP
Piezo2
Tubb3
100 µm
M
p<0.0001
244/245
217/219
10/202
86/94
Cre
control
Piezo2-cKO
N
responding neurons
WT
Piezo2-cKO
cheek brush
cheek pinch
O
Scn10a-Cre+/-;Ai95D
30 ΔF
P
Q
46/56

Figure S3. Intradental neurons respond to enamel damage, but not vibration or heating artifact, and validation of *Piezo2*-cKO and *Scn10a*-Cre strains. Related to Figure 3.

(A-C) Intradental HTMRs encode cutting of tooth enamel. (A) Example traces showing intradental neuron response to enamel cutting applied to individual molars. Stimuli used are indicated above the traces. Vertical scale bars, 40 ΔF. Horizontal scale bar, 20 s. (B) Example heatmap containing traces shown in (A). Scale bar, 40 s. (C) Bar graph showing proportion of cutting responsive intradental neurons identified by electrical stimulation. Plotted individual data points represent the proportion for each trial. Bar shows the mean and error bars indicate the SEM. n = 4 mice, comprising 70 total cells. Data for A-C were obtained from Ai95(RCL-GCaMP6f)-D (Ai95D) mice injected postnatally (P0) with AAV9-Cre.

(D-G) Intradental neurons do not respond to vibration applied to the intact tooth. (D) Example traces showing intradental neurons response to surface vibration of the intact tooth. Gray traces (3 examples of vibration responders) show that molar surface vibration induces transient responses in select non-intradental innervating neurons. Stimuli used are indicated above the traces. Vertical scale bars, 10 ΔF. Horizontal scale bar, 20 s. (E) Example heatmap related to traces shown in (H). Vibration responders were observed in non-intradental innervating neurons (did not respond to electrical or cold stimulation of molars). Neurons were grouped as intradental neurons (cells 1-16) or non-intradental vibration responders (cells 17-50). Stimuli used are indicated above the heatmap. Scale bar, 40 s. (F) Bar graph showing proportion of vibration responses/intradental neurons. Plotted individual data points represent the calculated proportion of intradental neurons that also respond to vibration. Bar shows the mean and error bars indicate the SEM. n = 3 mice, comprising 52 total intradental neurons. (G) Bar graph depicting the proportion of intradental neurons that respond to cold stimulation. Plotted individual data points represent the proportion of cold responsive/intradental neurons

identified in each experiment. Bar shows the mean, and error bars indicate the SEM. n = 3 mice, comprising 52 total cells. Data were obtained from Ai95(RCL-GCaMP6f)-D (Ai95D) mice injected postnatally (P0-P3) with AAV-Cre.

(H) Snapshot images showing occlusal surface structure of murine molars before and after friction and then cutting. Application of friction to m1 or m2 via a rounded "fluteless" carbide dental bur generates diminutive damage to the molar occlusal surface. Enamel cutting of m1 or m2 via a ¼ carbide dental bur produces shallow damage to the enamel occlusal surface as indicated by white flecks. Scale bar, 500 μm.

(I-K) Graphs depicting friction (I) and cutting (J) stimulation produce minimal temperature increases (<3ºC) in tooth pulp temperature as measured with a thermocouple implanted into the pulp bed while leaving the molar occlusal surface intact (see Methods for details). Traces show the average, shading indicates the SEM. n = 6 measurements per condition. Scale bars, 10 s. (K) Bar graph showing maximal change in temperature in the tooth pulp in response to stimuli. Plotted individual data points represent the calculated ΔTmax (°C) for each measurement. Bar shows the mean and error bars indicate the SEM. n = 6 (friction), n = 6 (cutting). $p > 0.05$ using unpaired t test.

(L) Representative ISH images for a single section of the trigeminal ganglion taken from Ai95D (top) or *Piezo2*-cKO; Ai95D mice (bottom) injected postnatally (P0-P3) with AAV9-Cre. Probes (left to right): *Piezo2* (red), *GCaMP* (green), *Tubb3* (cyan). Yellow arrow heads indicate *GCaMP*+ neurons, white arrow heads indicate *GCaMP*- neurons. In *Piezo2*-cKO; Ai95D mice white arrow heads correspond to neurons with cytoplasmic *Piezo2* without cKO. Scale bar, 100 μm.

(M) Bar graph showing the proportion of diffuse cytoplasmic *Piezo2* in TG neurons in Ai95D (top) or *Piezo2*-cKO; Ai95D mice. Cre expression was inferred by expression of GCaMP ISH signal. Plotted individual data points represent proportions scored within each section. Bar shows the mean and error bars indicate the SEM. n = 3 mice per group, with data taken from at least 3 sections, averaged per animal. $p < 0.0001$ or n.s. $p > 0.05$ using two-way ANOVA with Šídák's multiple comparisons test.

(N) Functional validation of AAV9 Cre mediated *Piezo2*-cKO. Bar graph showing in vivo functional imaging TG responses to cheek stimulation in WT versus *Piezo2*-cKO mice. Plotted individual data points represent responding neurons to either cheek brush or pinch. Bar shows the mean, and error bars indicate the SEM. n = 6 (WT), n = 6 (*Piezo2*-cKO). $p < 0.0001$ or n.s. = $p > 0.05$ using paired t test

(O-Q) *Scn10a*-Cre heterozygote enables functional identification of intradental HTMRs that respond to cutting

(O) Example traces showing cutting responses of intradental HTMRs in *Scn10a*-Cre+/-;Ai95D mice. Stimuli used are indicated above the traces. Vertical scale bars, 30 ΔF. Horizontal scale bar, 20 s. (P) Example heatmap containing traces shown in (O). Stimuli used are indicated above the heatmap. Scale bar, 20 s. (Q) Bar graph showing proportion of cutting responsive intradental neurons. Plotted individual data points represent the proportion for each trial. Bar shows the mean and error bars indicate the SEM. n = 3 mice, comprising 56 total cells.

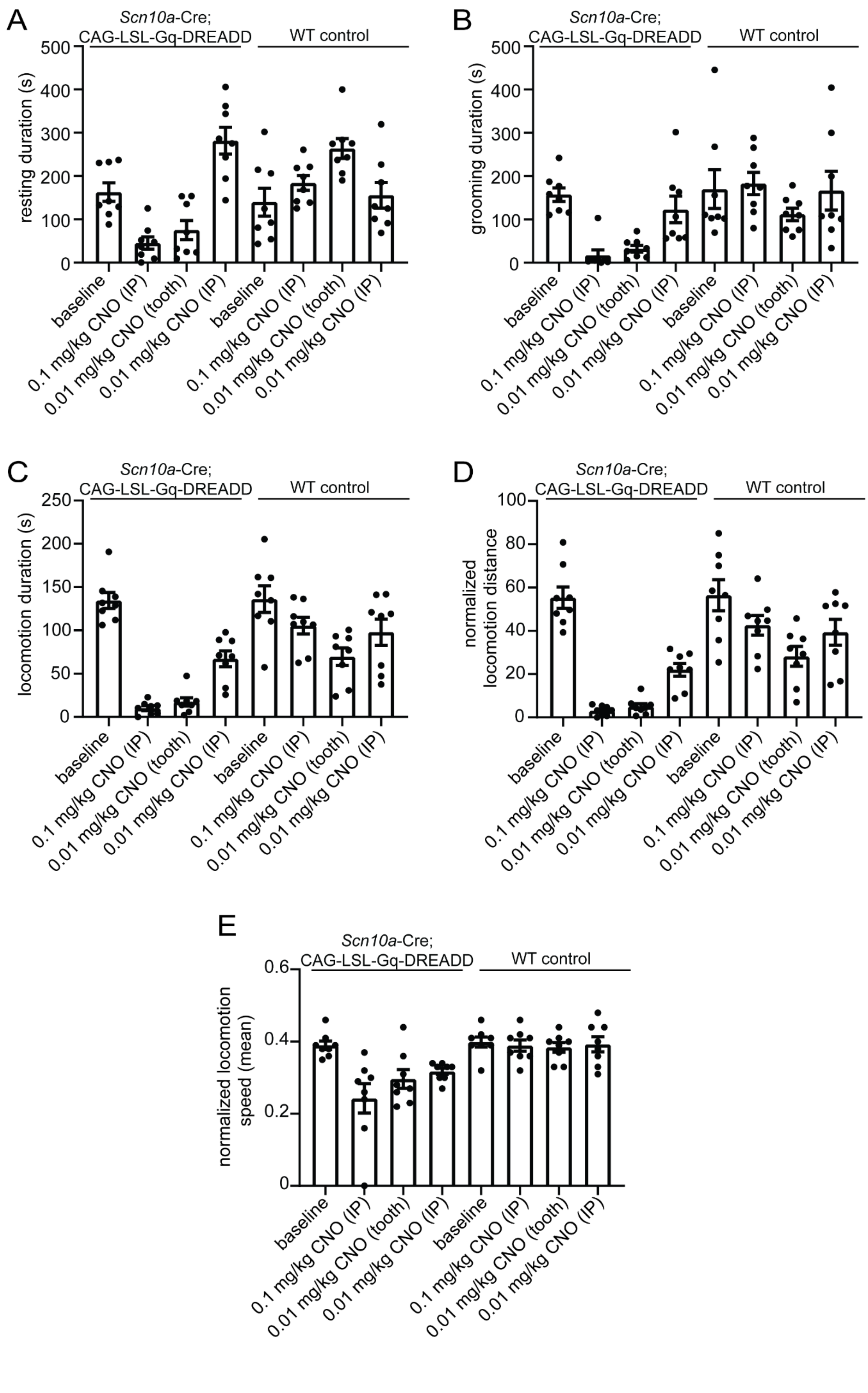

A
Scn10a-Cre;
CAG-LSL-Gq-DREADD
WT control
resting duration (s)
0
100
200
300
400
500
baseline
0.1 mg/kg CNO (IP)
0.01 mg/kg CNO (tooth)
0.01 mg/kg CNO (IP)
baseline
0.1 mg/kg CNO (IP)
0.01 mg/kg CNO (tooth)
0.01 mg/kg CNO (IP)
B
Scn10a-Cre;
CAG-LSL-Gq-DREADD
WT control
grooming duration (s)
0
100
200
300
400
500
baseline
0.1 mg/kg CNO (IP)
0.01 mg/kg CNO (tooth)
0.01 mg/kg CNO (IP)
baseline
0.1 mg/kg CNO (IP)
0.01 mg/kg CNO (tooth)
0.01 mg/kg CNO (IP)
C
Scn10a-Cre;
CAG-LSL-Gq-DREADD
WT control
locomotion duration (s)
0
50
100
150
200
250
baseline
0.1 mg/kg CNO (IP)
0.01 mg/kg CNO (tooth)
0.01 mg/kg CNO (IP)
baseline
0.1 mg/kg CNO (IP)
0.01 mg/kg CNO (tooth)
0.01 mg/kg CNO (IP)
D
Scn10a-Cre;
CAG-LSL-Gq-DREADD
WT control
normalized
locomotion distance
0
20
40
60
80
100
baseline
0.1 mg/kg CNO (IP)
0.01 mg/kg CNO (tooth)
0.01 mg/kg CNO (IP)
baseline
0.1 mg/kg CNO (IP)
0.01 mg/kg CNO (tooth)
0.01 mg/kg CNO (IP)
E
Scn10a-Cre;
CAG-LSL-Gq-DREADD
WT control
normalized locomotion
speed (mean)
0
0.2
0.4
0.6
baseline
0.1 mg/kg CNO (IP)
0.01 mg/kg CNO (tooth)
0.01 mg/kg CNO (IP)
baseline
0.1 mg/kg CNO (IP)
0.01 mg/kg CNO (tooth)
0.01 mg/kg CNO (IP)

Figure S4. Quantification of resting, grooming, and locomotion behavior from *Scn10a*-Cre;CAG-LSL-Gq-DREADD and WT control mice. Related to Figure 4.

(A) Bar graph depicting resting duration across conditions. Plotted individual data points represent cumulative values for individual animals. Bar shows the mean, and error bars indicate the SEM (see Table S1B for statistics).

(B) Bar graph depicting grooming duration across conditions. Plotted individual data points represent cumulative values for individual animals. Bar shows the mean, and error bars indicate the SEM (see Table S1C for statistics).

(C) Bar graph depicting locomotion duration across conditions. Plotted individual data points represent cumulative values for individual animals. Bar shows the mean, and error bars indicate the SEM (see Table S1D for statistics).

(D) Bar graph depicting normalized locomotion distance across conditions. Plotted individual data points represent cumulative values for individual animals. Bar shows the mean, and error bars indicate the SEM (see Table S1E for statistics).

(E) Bar graph depicting mean normalized locomotion speed across conditions. Plotted individual data points represent the mean for individual animals. Bar shows the mean, and error bars indicate the SEM (see Table S1F for statistics).

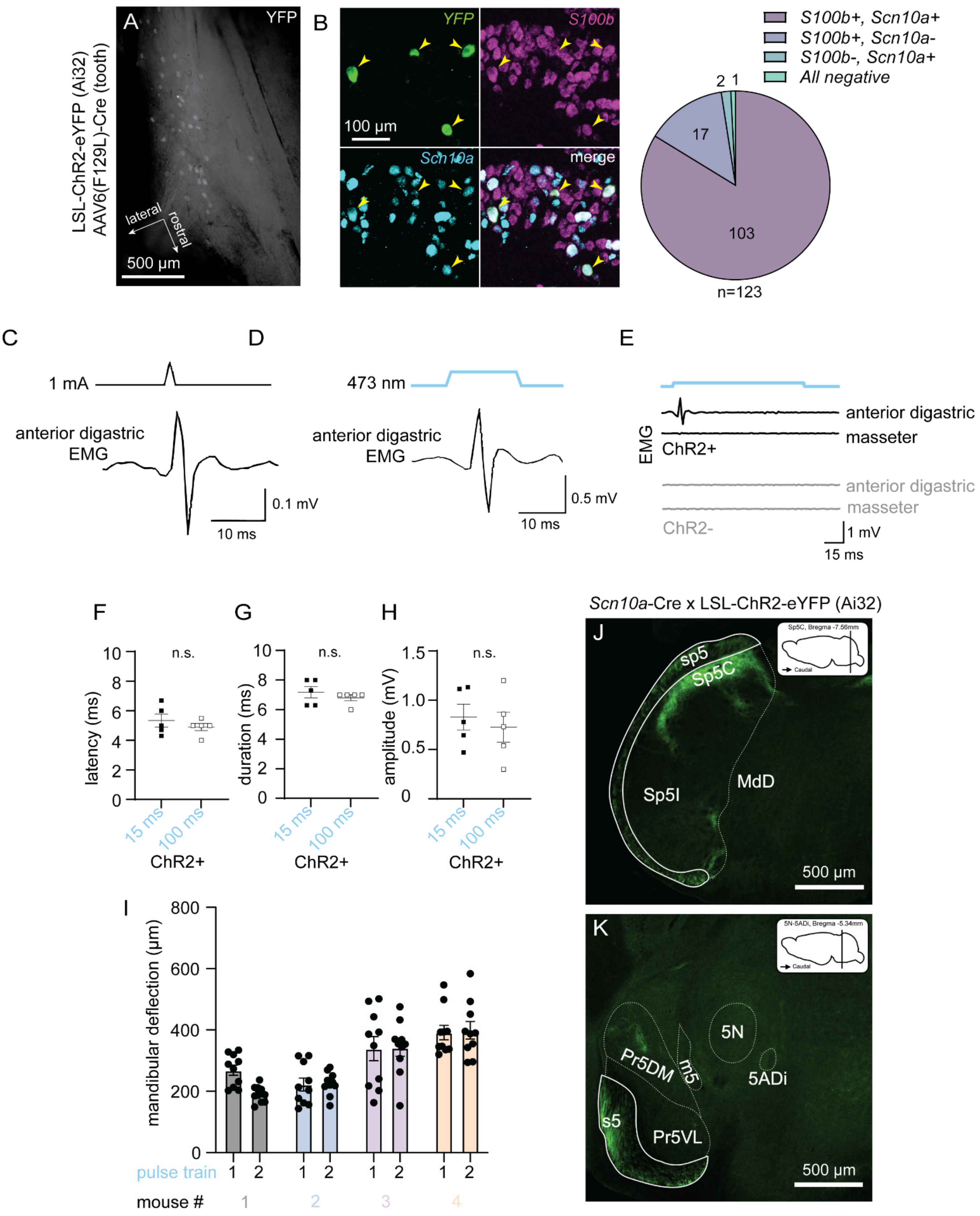

LSL-ChR2-eYFP (Ai32)
AAV6(F129L)-Cre (tooth)
A
YFP
lateral
rostral
500 µm
B
YFP
S100b
Scn10a
merge
100 µm
S100b+, Scn10a+
S100b+, Scn10a-
S100b-, Scn10a+
All negative
2 1
17
103
n=123
C
1 mA
anterior digastric
EMG
0.1 mV
10 ms
D
473 nm
anterior digastric
EMG
0.5 mV
10 ms
E
EMG
anterior digastric
masseter
ChR2+
anterior digastric
masseter
ChR2-
1 mV
15 ms
F
G
H
n.s.
latency (ms)
duration (ms)
amplitude (mV)
15 ms
100 ms
ChR2+
I
mandibular deflection (µm)
pulse train
mouse #
Scn10a-Cre x LSL-ChR2-eYFP (Ai32)
J
Sp5C, Bregma -7.56mm
Caudal
sp5
Sp5C
Sp5I
MdD
500 µm
K
5N-5ADi, Bregma -5.34mm
Caudal
5N
Pr5DM
m5
5ADi
s5
Pr5VL
500 µm

Figure S5. Electromyography and jaw deflection elicited by activation of intradental neurons. *Scn10a*+ projections directly target the spinal trigeminal nucleus, not the trigeminal motor nuclei. Related to Figure 5.

(A) Example image of the dorsal view of the excised ganglion showing ChR2-eYFP positive intradental neurons in the TG induced by AAV-Cre injection into the ipsilateral molars. Scale bar, 500 μm.

(B) Left: Representative ISH images for a single section of the trigeminal ganglion showing labeled YFP+ intradental neurons taken from an Ai32 (Cre-dependent ChR2-YFP) mouse receiving AAV-Cre tooth injections. Probes: *YFP* (top left), *S100b* (top right), *Scn10a* (bottom left), merge (bottom right). Yellow arrow heads indicate *YFP*+ intradental neurons. Scale bar, 100 μm. Right: Pie chart representing counts of *YFP*+ intradental neurons expressing *Scn10a* and/or *S100b*. n = 4 mice, comprising 123 total *YFP*+ neurons.

(C) Example trace of anterior digastric muscle EMG elicited by electrical stimulation of inferior alveolar nerve (1 mA, 0.1 ms, 1 Hz electrical stimulation). Experiment was repeated in n = 3 mice. Vertical scale bar, 0.1 mV. Horizontal scale bar, 10 ms.

(D) Example trace of anterior digastric muscle EMG elicited by optogenetic activation of ipsilateral intradental neurons (15 ms, 1 Hz, 43 nm laser activation). Experiment was repeated in n = 3 mice. Vertical scale bar, 0.5 mV. Horizontal scale bar, 10 ms.

(E) Optogenetic activation (100 ms duration) induces anterior digastric muscle activity when channelrhodopsin-2 is expressed in intradental neurons, with no activity observed in the masseter muscle. Control ChR2- group shows no changes in muscle activity for either the anterior digastric or masseter muscles. Experiment was repeated in n = 5 mice. Vertical scale bar, 1 mV. Horizontal scale bar, 15 ms.

(F-H) Graph showing the (F) latency, (G) duration, and (H) amplitude of the digastric muscle activity elicited 15 ms or 100 ms optogenetic activation depicting average +/- SD for each condition. (F) Latency: 15 ms: 5.2 +/- 1.3 ms vs. 100 ms: 4.8 +/- 0.8 ms. (G) Duration: 15 ms: 6.6 +/- 0.6 ms vs. 100 ms: 6.7 +/- 0.6 ms. (H) Amplitude: 15 ms: 0.97 +/- 0.3 mV vs. 100 ms: 0.93 +/- 0.2 mV. For all conditions n = 5 mice and $p > 0.05$ using unpaired t test.

(I) Bar graph depicting the amplitude of mandibular deflections in response to a blue-light pulse trains (10 pulses, 470nm, 1s), from data shown in Figure 4F, G. Plotted data points represent the measurements from individual mandibular deflections during pulse trains. Bar shows the mean and error bars indicate the SEM. n = 4 mice, comprising 160 measurements. $p > 0.05$ using unpaired t test.

(J-K) Representative images of coronal sections where eYFP fluorescence marks *Scn10a*+ nerve fibers. Sagittal outlines from the Allen Mouse Brain Atlas Sections indicate the corresponding coronal planes. (J) Coronal section at bregma -7.56 mm showing the caudal part of the spinal trigeminal nucleus (Sp5C). Scale bar, 500 µm. (K) Coronal section of the motor trigeminal nucleus (5N) or the anterior digastric part of the motor trigeminal nucleus (5ADi) at bregma -5.34 mm. n = 3 mice were utilized for anatomical mapping and confirmation of intradental nerve fiber projections. Scale bar, 500 µm. Abbreviations (Allen Brain Atlas): Spinal trigeminal tract (sp5); Spinal trigeminal nucleus, caudal part (Sp5C); Spinal trigeminal nucleus, interpolar part (Sp5I); Medullary reticular nucleus, dorsal part (MdD); Sensory root of trigeminal nerve (s5); Principal sensory trigeminal nucleus, dorsomedial part (Pr5DM); Principal sensory trigeminal nucleus, ventrolateral part (Pr5VL); Motor root of the trigeminal nerve (m5); Motor trigeminal nucleus (5N); Motor trigeminal nucleus, anterior digastric part (5ADi).
